# Population Diversity, Dynamics, and Differentiation of Wheat Stripe Rust Pathogen *Puccinia striiformis* f. sp. *tritici* From 2010 to 2017 and Comparison With 1968 to 2009 in the United States

**DOI:** 10.3389/fmicb.2021.696835

**Published:** 2021-07-22

**Authors:** Qing Bai, Anmin Wan, Meinan Wang, Deven R. See, Xianming Chen

**Affiliations:** ^1^Department of Plant Pathology, Washington State University, Pullman, WA, United States; ^2^Wheat Health, Genetics, and Quality Research Unit, United States Department of Agriculture, Agricultural Research Service, Pullman, WA, United States

**Keywords:** population genetics, *Puccinia striiformis* f. sp. *tritici*, SSR markers, wheat stripe rust, fungal pathogen evolution

## Abstract

Stripe rust, caused by *Puccinia striiformis* f. sp. *tritici* (*Pst*), is a serious disease on wheat in the United States, especially after 2000. In the present study, 2,247 *Pst* isolates collected over all stripe rust epidemiological regions in the United States from 2010 to 2017 were genotyped at 14 simple sequence repeat (SSR) loci to investigate the population diversity, dynamics, and differentiation. A total of 1,454 multilocus genotypes (MLGs) were detected. In general, the populations in the west (regions 1–6) had more MLGs and higher diversities than the populations in the east (regions 7–12). The populations of 2010 and 2011 were more different from the other years. Genetic variation was higher among years than among regions, indicating the fast changes of the population. The divergence (*Gst*) was bigger between the west population and east population than among regions within either the west or east population. Gene flow was stronger among the regional populations in the east than in the west. Clustering analyses revealed 3 major molecular groups (MGs) and 10 sub-MGs by combining the genotypic data of 2010–2017 isolates with those of 1968–2009. MG1 contained both 1968–2009 isolates (23.1%) and 2010–2017 isolates (76.9%). MG2 had 99.4% of isolates from 1968–2009. MG3, which was the most recent and distinct group, had 99.1% of isolates from 2010–2017. Of the 10 sub-MGs, 5 (MG1-3, MG1-5, MG3-2, MG3-3, and MG3-4) were detected only from 2011 to 2017. The SSR genotypes had a moderate, but significant correlation (*r* = 0.325; *p* < 0.0001) with the virulence phenotype data. The standard index values of association (rbarD = 0.11) based on either regional or yearly populations suggest clonal reproduction. This study indicated high diversity, fast dynamics, and various levels of differentiation of the *Pst* population over the years and among epidemiological regions, and the results should be useful for managing wheat stripe rust.

## Introduction

The fungal pathogen *Puccinia striiformis* Westend. f. sp. *tritici* Erikss. (*Pst*) causing stripe (yellow) rust is one of the most destructive pathogens threatening global wheat production (Chen, 2005, 2020; Wellings, 2011). The United States is one of the most serious stripe rust epidemic countries. In the United States, wheat stripe rust was first recognized in 1915, but its epidemic mainly occurred in the west, especially the Pacific Northwest and California before 2000 (Carleton, 1915; Line and Qayoum, 1992; Line, 2002). Since 2000, however, wheat stripe rust has become more frequent and caused severe epidemics across the continental United States including the states east of the Rocky Mountains (Chen, 2005, 2007; Chen et al., 2010).

To understand the disease epidemiology and pathogen variation, a series of studies have been done on the *Pst* pathogen from virulence testing, population structure, reproductive system, mutation, somatic recombination to genome sequencing, and functional genomics (Chen et al., 2002, 2010; Wan and Chen, 2012, 2014; Cheng and Chen, 2014; Wang and Chen, 2015; Wang et al., 2015; Cheng et al., 2016; Wan et al., 2016; Xia et al., 2016, 2018a,b, 2020; Lei et al., 2017; Liu et al., 2017, 2021; Li et al., 2019b, c, 2020). Previous studies on virulence and molecular analyses support asexual reproduction of *Pst* in the United States Pacific Northwest (Cheng and Chen, 2014; Liu et al., 2021). Wan et al. (2014) identified higher diversity and higher genetic differentiation in the west population than the east population of the United States using simple sequence repeat (SSR) markers. Similarly, Xia et al. (2016) also found that the west population was more diverse than the east population using secreted-protein single-nucleotide polymorphism (SP-SNP) markers. However, these studies, which used a relatively small number of isolates collected in 2010 and/or 2011, are considered as pilot studies to demonstrate the usefulness of different types of molecular markers for a snapshot of the population structure. Because of this reason, these studies could not provide long-term dynamics of the *Pst* population. Using both SSR and SP-SNP markers, more than 1,000 isolates representing collections from 1968 to 2009 were characterized (Liu et al., 2021). This study of *Pst* isolates from over 40 years identified several events of introduction and a trend of the *Pst* populations changing from relatively simple to complex. However, the isolates collected since 2010 have been tested only for virulence and identified to races^1^. These collections have not been molecularly characterized. It is not clear whether the US *Pst* population has undergone any major genetic changes since 2010.

In 2010, wheat stripe rust caused a widespread epidemic across the United States in the recorded history, resulting in large-scale application of foliar fungicides, especially in the Great Plains and the western states (Wan and Chen, 2016). In 2011 and 2012, stripe rust continued causing severe damage in the Pacific Northwest (Chen, 2014; Wan et al., 2016). Furthermore, the stripe rust epidemics in both 2015 and 2016 were severe, causing country-wide yield losses of 8.2 and 5.6%, respectively (Chen and Kang, 2017; Chen, 2020). In addition to the favorable weather conditions, the high number of new races and widespread predominant races identified in these years were attributed at least partially to the epidemics (Wan and Chen, 2012, 2016; Wan et al., 2016, 2017). However, it was not clear whether the pathogen population had significant changes in genetic structures. Therefore, this study is conducted to characterize the population diversity and dynamics of *Pst* from 2010–2017 and compare the populations in these years with those of 1969–2009 characterized by Liu et al. (2021) using the selected SSR markers to provide the long-term dynamics of the pathogen in the United States.

## Materials and Methods

### Sample Collection and Urediniospore Multiplication

During the growing seasons from 2010 to 2017, stripe rust samples were collected from commercial wheat fields, breeding nurseries, variety trials, and disease screening nurseries across all 12 regions of stripe rust epidemiology throughout the United States as previously described (Chen et al., 2010) (Table 1 and Supplementary Figure 1). A total of 2,247 isolates were obtained, and the urediniospores were multiplied for DNA extraction. In brief, each leaf sample was washed with water, placed on a wet filter paper in a Petri dish, and incubated at 4°C overnight. The fresh urediniospores were transferred with a fine brush onto two-leaf-stage seedlings of wheat cultivar “Nugaines” that is susceptible at the seedling stage to all *Pst* races identified thus far in the United States. The inoculated plants were incubated in a dew chamber at 10°C for 24 h without light and grown in a growth chamber at a diurnal cycle gradually changing from 4°C at 2:00 AM to 20°C at 2:00 PM with 8-h dark/16-h light corresponding to the low/high temperature periods (Chen et al., 2002, 2010). To prevent cross-contamination, plants inoculated with different isolates were separated with plastic booths. Urediniospores were vacuum collected with a custom-made glass collector and kept in a desiccator for later use.

**TABLE 1:** The numbers of *Puccinia striiformis* f. sp. *tritici* isolates obtained from 12 epidemiological regions in the United States from 2010 to 2017.

	Number of isolates														
		West							East						
Year	Subtotal	R1^a^	R2	R3	R4	R5	R6	Subtotal	R7	R8	R9	R10	R11	R12	Subtotal
2010	322	94	16	6	4	21	65	206	60	10	22	1	12	11	116
2011	319	146	14	54	0	19	47	280	14	8	3	1	10	3	39
2012	201	87	2	2	2	18	12	123	26	9	12	3	19	9	78
2013	353	167	3	0	22	44	24	260	33	21	1	17	15	6	93
2014	315	161	17	9	1	45	58	291	19	2	1	1	0	1	24
2015	220	108	13	4	5	18	25	173	23	7	8	6	2	1	47
2016	290	103	14	9	9	18	17	170	25	11	24	19	16	25	120
2017	227	107	12	3	1	24	17	164	14	5	12	0	14	18	63
Total	2,247	973	91	87	44	207	265	1,667	214	73	83	48	88	74	580

*^a^Region 1 (R1) = eastern Washington, northeastern Oregon, and northern Idaho; R2 = western Montana; R3 = southern Idaho, southeastern Oregon, northern Nevada, northern Utah, western Wyoming, and western Colorado; R4 = western Oregon and northern California; R5 = northwestern Washington; R6 = central and southern California, Arizona, and western New Mexico; R7 = Texas, Louisiana, Arkansas, Oklahoma, and eastern New Mexico; R8 = Kansas, Nebraska, and eastern Colorado; R9 = South Dakota, North Dakota, Minnesota, and eastern Montana; R10 = Mississippi, Alabama, Florida, Georgia, South Carolina, North Carolina, Tennessee, and Kentucky; R11 = Missouri, Illinois, Indiana, Iowa, Wisconsin, and Michigan; R12 = Virginia, West Virginia, Ohio, Maryland, Pennsylvania, and New York (Chen et al., 2010).*

### Genomic DNA Extraction

Total genomic DNA was extracted from dried urediniospores following a universal and rapid salt-extraction method for high-quality genomic DNA (Aljanabi and Martinez, 1997) with modifications for *Pst* (Justesen et al., 2002). A mixture of ∼20 mg spores and 200 mg sand in a 1.1-mL tube was ground by vortexing for 2 min and added with 500 μL 2x cetyltrimethylammonium bromide buffer (1.4 M NaCl, 100 mM Tris–HCl pH 8.0, and prewarmed to 65°C). After mixing, the tube was incubated in a water bath at 65°C for 60 min with gently inverting every 10 min, added with 400 μL chloroform: isopentyl alcohol (24:1), and centrifuged for 15 min at 4,000 revolutions/min at 4°C in an Allegra 25R centrifuge (Beckman Coulter Inc., Brea, CA, United States). The 500 μL supernatant was transferred to a new tube and added with 500 μL isopropanol. The mixture was centrifuged for 15 min, and the DNA pellet was air-dried and resuspended with 100 μL TE buffer (containing 20 μg mL^–1^ RNase A). The tube was incubated at 37°C for 2 h to completely dissolve the DNA pellet. The concentration of the DNA stock solution was determined using an ND-1000 spectrophotometer (Bio-Rad, Hercules, CA, United States), and the quality was checked in a 0.8% agarose gel. A work solution of 5 ng μL^–1^ was made from the stock solution by adding with sterile deionized water for use as a DNA template in polymerase chain reactions (PCRs).

### SSR Markers and PCR Amplification

Fourteen pairs of SSR primers were selected based on their codominant polymorphisms among *Pst* isolates in previous studies (Cheng et al., 2012, 2016; Cheng and Chen, 2014; Sharma-Poudyal et al., 2020; Liu et al., 2021). They were CPS02, CPS04, CPS08, and CPS13 (Chen et al., 2009); PstP001, PstP002, PstP003, PstP005, PstP006, and PstP029 (Cheng et al., 2012); and RJ18, RJ20, RJ21, and RJ8N (Enjalbert et al., 2002; Bahri et al., 2009a). These markers, except PstP001 and PstP005, were previously used by Liu et al. (2021) to characterize the *Pst* collections from 1968 to 2009. They used two additional markers (PstP004 and PstP033). For unresolved issues, PstP004 and PstP033 did not produce fragments in many isolates in the preliminary tests of the present study, and therefore, these two markers were replaced with PstP001 and PstP005. The sequences, annealing temperatures, and amplified fragments of the primers are provided in Supplementary Table 1. To use fluorescence for detecting PCR products, an M13 tag (5′-CACGACGTTGTAAAACGAC) was added to the 5′ end of each forward primer (Schuelke, 2000). For each SSR locus, the forward primer was labeled with black, green, or blue florescent dye, with the red fluorescent dye used for the size marker. SSR loci with the same or close allele sizes were labeled with different florescent dyes to achieve the maximum possible number of the loci per run in the sequencer.

Amplification of PCR was performed in a Bio-Rad iCycler (Bio-Rad, Hercules, CA, United States) following the protocol described in previous studies (Cheng et al., 2012; Cheng and Chen, 2014). Each reaction (12 μL) contained 1.2 μL of 10x reaction buffer with 15 mM MgCl_2_, 0.96 μL 2.5 mM dNTP, 0.12 μL of 5 μM forward primer, 0.6 μL 5 μM reverse primer, 0.24 μL of 5 μM M13 universal primer, 0.2 μL 5 U/μL *Taq* polymerase, 4 μL DNA (total 20 ng), and 4.68 μL sterile ddH_2_O. The amplification cycles and conditions were 94°C for 5 min for initial denaturation; 42 cycles of 94°C for 30 s, 45–54°C for 30 s depending on primers, and 72°C for 45 s; and 7 min of final extension at 72°C. The sizes of the PCR products were estimated using capillary electrophoresis on an ABI3730 Genotyper (Applied Biosystems, Foster City, CA, United States). The internal molecular weight standard for ABI3730 was Genescan 445-LIZ (Applied Biosystems). Allele sizes in base pairs were scored and analyzed using software GeneMarker V2.2 (Softgenetics, State College, PA, United States).

### Analyses of Multilocus Genotypes and Genotypic Diversity

If isolates in the present study have identical alleles across all 14 SSR loci, they were assigned to the same multilocus genotype (MLG). The MLGs were named in continuation with the previous MLGs identified from the isolates of 1968 to 2009. As 12 SSR markers were commonly used in the present study and the previous study (Liu et al., 2021), we used the 12 markers to find isolates that have identical alleles with previously designated MLGs and used the previous MLGs to designate the isolates in the present study. For isolates that were identical at the 12 common marker loci, but different at the two additional marker loci used in the present study, they could be numbered the same MLG plus extension numbers to indicate the differences. Actually, we did not encounter any of such cases in the present study.

As *Pst* is a dikaryotic fungus at the uredinial stage, each isolate was scored for two alleles to determine homozygous or heterozygous for each SSR locus using GeneMarkerV2.2 (Softgenetics). The data file generated by GeneMarker was converted to a “csv” file to be analyzed with the R package. The sufficiency of markers to describe the population structure was assessed through the detection of MLGs plotted against the number of loci, and a graph was generated using the “*poppr*” package in the R program (Kamvar et al., 2014). The genetic relationships among individual MLGs were analyzed based on Bruvo’s distance and presented by a minimum spanning network in “*poppr*.” Genotypic diversity was determined by estimating both genotypic richness (the number of observed MLGs) and evenness (the distribution of genotype abundance). Stoddart and Taylor’s index and Shannon’s diversity index for MLG diversity were calculated as *G* = 1/Σ*P*_i_^2^ and *H* = Σ*P*_i_ln*P*_i_, respectively, where *P*_i_ is the observed frequency of the *i*th MLG in a population (Shannon, 1948, 2001; Stoddart and Taylor, 1988). Moreover, evenness (*E*_5_) (values of *E*_5_ ranging from 0 to 1, with low values indicating that a certain genotype dominates in the collection of isolates) (Grünwald et al., 2003) and Nei’s unbiased gene diversity that is also referred as average heterozygosity (*H*_exp_, corrected for sample size) (Nei, 1978) were calculated for each region (R1–R12), the west (R1–R6), and east (R7–R12) regions, and each year (2010–2017).

### Spatial and Temporal Population Variation, Differentiation, and Phylogenetic Relationships

Analysis of molecular variance (AMOVA), which allows the hierarchical partitioning of genetic variation within and between regions and years (Excoffier et al., 1992), was conducted by defining the *Pst* isolates based on stripe rust epidemiological regions in the United States from 2010 to 2017 using function “poppr.amova” in the *poppr* R program version 4.0.3 (Kamvar et al., 2014; Meirmans and Liu, 2018). Population differentiation measured by the standardized coefficient of gene differentiation (*Gst*) (Hedrick, 2005) among the 12 regions and 8 years was analyzed using the software GenAlEx 6.503 (Peakall and Smouse, 2006), and the *Gst* values of the 12 regional populations were also used for generating a migration network using the “*diversity*” package in the R program to visualize the gene flow patterns among the epidemiological regions. To further investigate the population differentiation at the spatial and temporal levels, phylogenetic trees were generated based on Nei’s genetic distance for the 12 epidemiological regions and 8 years in bootstrap analysis with 1,000 replicates using the function “aboot” in the “*poppr*” program.

### Identification of Molecular Groups

To identify putative clusters of genetically related isolates in the present study (2,247 isolates) in comparison with the 1,083 isolates from 1968 to 2009 (Liu et al., 2021), hierarchical clustering analysis based on the 12 SSR markers (CPS02, CPS04, CPS08, CPS13, RJ18, RJ20, RJ21, RJ8N, PstP002, PstP003, PstP006, and PstP029), which were commonly used by Liu et al. (2021) and in the present study, was conducted using the dissimilarity values and the “ward.D2” method with the “hclust” function in the R stats 4.0.3 program (Murtagh and Legendre, 2014). The parameters for hierarchical cluster analysis were the same as previously described (Sharma-Poudyal et al., 2020). The discriminant analysis of principal components (DAPC), a no model–based method developed and implemented in the “*adegenet*” R package, was performed to generate a scatterplot about the membership probability of each isolate in the corresponding molecular groups (MGs) (Jombart, 2008; Jombart et al., 2010). To assess how the temporal populations differ from each other, the DAPC was also performed based on the yearly populations. The DAPC was carried out for all loci, and an α score optimization was used to determine the number of principal components to retain.

### Reproduction Mode

The mode of reproduction was assessed by evaluating observed linkage among SSR loci against expected distributions from permutation using the index of association (IA) (Brown et al., 1980a, b; Goss et al., 2014). Unfortunately, IA has been reported to increase with the number of loci and thus is not suitable for comparisons across studies (Agapow and Burt, 2001). To remedy this, adjusted/standard IA (rbarD) has been introduced to force the index to lie between 0 (linkage equilibrium) and 1 (full disequilibrium) (Agapow and Burt, 2001). Therefore, in the present study, rbarD was calculated using the “*poppr*” program, and the null hypothesis rbarD = 0 was tested with 1,000 permutations (Kamvar et al., 2014). If an observed rbarD value was outside the distribution expected from unlinked loci at *p* < 0.001, the population was considered clonal (Goss et al., 2014; Kamvar et al., 2014).

### Determination of Correlation Between Molecular Genotypes and Virulence Phenotypes

The 2,221 of 2,247 isolates were previously studied for virulence and identified to races^2^. Mantel tests were conducted for the correlation coefficient between the molecular data and virulence data. The genetic distance matrix based on SSR markers among all isolates was obtained using function “genetic_distance” in the “*gstudio*” package, and the mantel function was obtained using the “*vegan*” package in the R Program 4.0.3 (Mantel, 1967; Dyer et al., 2004; Legendre and Legendre, 2012).

## Results

### The Number, Frequency, and Distribution of MLGs

The marker sufficiency test found that 97.8% (1,422 of 1,454) of the MLGs could be identified with 13 markers (CPS02, CPS04, CPS08, CPS13, PstP001, PstP002, PstP003, PstP005, PstP006, RJ18, RJ20, RJ21, and RJ8N), and the inclusion of one more marker (PstP029) did not significantly change the number of MLGs (Supplementary Figure 2). The result indicated that the 14 markers were sufficient for studying the genetic structure of *Pst* population.

With the 14 SSR markers, 1,454 MLGs were identified from the 2,247 isolates of the overall US *Pst* population from 2010 to 2017. The allelic genotypes of each of the 2,247 isolates at the 14 marker loci and their assigned MLG are provided in Supplementary Table 2, and the marker alleles for each of the 1,454 MLGs with the number of isolates are provided in Supplementary Table 3. Of the 1,454 MLGs, 1,263 each had only 1 isolate, 173 had 2–8 isolates, and 18 had 10–108 isolates. The 18 predominant MLGs with 10 or more isolates were MLG2026 (108 isolates), MLG500 (81 isolates), MLG1055 (27 isolates), MLG671 (26 isolates), MLG1099 (24 isolates), MLG1743 (22 isolates), MLG1989 (17 isolates), MLG1623 (16 isolates), MLG2018 (15 isolates), MLG1082 (13 isolates), MLG1550 (13 isolates), MLG27 (12 isolates), MLG650 (12 isolates), MLG1355 (12 isolates), MLG2023 (12 isolates), MLG989 (11 isolates), MLG1106 (11 isolates), and MLG909 (10 isolates). The MLGs detected in two or more regions, as well as those with two or more isolates but detected in only one region, are presented in Supplementary Table 4.

Among the 12 regions, R1 had the highest number of MLGs (730) and the highest number of private MLGs (639), whereas R4 had the lowest number (17) of MLGs and the lowest number (7) of private MLGs (Table 2). These results were related to the highest number of isolates (973) in R1 and the lowest number of isolates (22) in R4. However, when the number of isolates was considered, these two regions had relatively low MLG/isolate (*g*/*N*) ratios (0.75 and 0.77, respectively), whereas R8, R10, and R12 had the highest *g*/*N* ratios (0.92, 0.94, and 0.95, respectively). Across the west regions (R1–R6), 1,118 MLGs were identified from 1,667 isolates, whereas 420 MLGs were identified from 580 isolates across the east region (R7–R12). The east region had a relatively high *g*/*N* ratio (0.72) compared to the west region (0.67). Overall, the whole country had a *g*/*N* ratio of 0.65; fewer than two samples needed to identify one MLG.

**TABLE 2:** Numbers of *Puccinia striiformis* f. sp. *tritici* isolates, numbers of multilocus genotypes (MLGs), numbers and frequencies (%) of private MLGs, *g*/*n* values, Stoddart and Taylor’s MLG diversity (G) values and confidence intervals (CIs), Shannon–Wiener index values of MLG diversity (H) and confident intervals (CIs), evenness (*E*_5_), Nei’s unbiased gene diversity (*H*_*exp*_) values over 14 SSR loci, and standardized index of association (rbarD) values and probability (*p*) values for indication of reproduction mode in collections of 12 epidemiological regions (R1–R12) in the United States.

Region	No. of isolates (n)	No. of MLGs (g)	*g/n*	No. and freq. (%) of private MLGs	Stoddart and Taylor’s MLGs diversity (G) (CI)^a^	Shannon–Wiener index of MLG diversity (H) (CI)^b^	*E*_5_^c^	*H*_exp_^d^	Standardized index of association (rbarD)^e^	*p-*value (rbarD)
R1	973	730	0.75	639 (87.5)	209.13 (166.33, 251.93)	6.29 (6.22, 6.36)	0.39 (0.29, 0.50)	0.40	0.09	<0.001
R2	91	76	0.84	55 (72.4)	49.58 (43.49, 55.68)	4.19 (4.03, 4.36)	0.74 (0.61, 0.88)	0.44	0.12	<0.001
R3	109	84	0.77	47 (56.0)	32.02 (26.88, 37.16)	4.13 (3.92, 4.36)	0.51 (0.35, 0.66)	0.39	0.19	<0.001
R4	22	17	0.77	7 (41.2)	12.74 (11.73, 13.74)	2.71 (2.40, 3.02)	0.83 (0.70, 0.97)	0.42	0.17	<0.001
R5	207	158	0.76	127 (80.4)	64.63 (54.72, 74.54)	4.78 (4.64, 4.93)	0.54 (0.42, 0.65)	0.39	0.09	<0.001
R6	265	164	0.62	125 (76.2)	57.05 (49.75, 64.34)	4.68 (4.55, 4.82)	0.52 (0.43, 0.61)	0.44	0.18	<0.001
*West*	1,667	*1,118*	*0.67*	*1,000 (89.4)*	*162.2 (125.52, 198.87)*	*6.13 (6.44, 6.58)*	*0.24 (0.18, 0.30)*	*0.41*	*0.10*	<*0.001*
R7	214	143	0.67	98 (68.5)	37.79 (30.67, 44.90)	4.54 (4.37, 4.70)	0.40 (0.27, 0.52)	0.45	0.13	<0.001
R8	73	67	0.92	50 (74.6)	59.88 (56.48, 63.28)	4.16 (4.01, 4.31)	0.93 (0.87, 0.99)	0.44	0.07	<0.001
R9	83	71	0.86	49 (69.0)	56.01 (51.50, 60.52)	4.18 (4.02, 4.33)	0.86 (0.77, 0.95)	0.44	0.12	<0.001
R10	48	45	0.94	30 (66.7)	41.14 (39.04, 43.25)	3.77 (3.59, 3.96)	0.94 (0.87, 1.01)	0.41	0.11	<0.001
R11	88	77	0.88	53 (68.8)	54.54 (48.53, 60.54)	4.24 (4.07, 4.40)	0.78 (0.67, 0.9)	0.43	0.08	<0.001
R12	74	70	0.95	47 (67.1)	62.23 (58.24, 66.22)	4.21 (4.07, 4.35)	0.92 (0.85, 0.99)	0.43	0.06	<0.001
*East*	*580*	*420*	*0.72*	*327 (77.9)*	*97.6 (75.88, 119.25)*	*5.24 (5.54, 5.76)*	*0.34 (0.24, 0.44)*	*0.44*	*0.10*	<*0.001*
Total	2,247	1,454	0.65	1,327 (91.3)	192.64 (162.61, 222.65)	6.71 (6.65, 6.77)	0.23 (0.19, 0.27)	0.43	0.11	<0.001

*^a^Stoddart and Taylor’s Index of MLG diversity and CI (Stoddart and Taylor, 1988) calculated using the “poppr” package in the R program 4.3. ^b^Shannon–Wiener Index of MLG diversity and confidence interval (Shannon, 1948) calculated using the “poppr” package in the R program 4.3. ^c^Evenness, E_5_. (Grünwald et al., 2003). ^d^Nei’s unbiased gene diversity (Nei, 1978) over 14 SSR loci. ^e^Standardized index of association, rbarD. p < 0.001 indicates significant disequilibrium due to linkage among loci, indicating populations are clonal (Agapow and Burt, 2001).*

At the temporal level, the number of MLGs varied from year to year, ranging from 99 in 2010 to 290 in 2013 (Table 3). The MLG/isolate (*g*/*N*) ratio ranged from 0.31 in 2010–0.92 in 2017. The ratios in 2010 and 2011 (0.31–0.44) were lower than those of 2012–2017 (0.80–0.92). However, the frequencies of new MLGs were higher in 2010–2013 (91.1–95%) than those of 2014–2017 (86.8–89%). The frequency of private MLGs, detected only in that particular year, was the highest in 2010 (90.9%) and the lowest in 2015 (82.5%).

**TABLE 3:** Numbers of *Puccinia striiformis* f. sp. *tritici* isolates, numbers of multilocus genotypes (MLGs), numbers and frequencies (%) of new and private MLGs, Stoddart and Taylor’s MLG diversity (G) values and confidence intervals (CIs), Shannon–Wiener index values of MLG diversity (H) and confident intervals (CIs), evenness (*E*_5_), Nei’s unbiased gene diversity (*H*_*exp*_) values over 14 SSR loci, and standardized index of association (rbarD) values and probability (*p*) values for indication of reproduction mode in collections of 8 years from 2010 to 2017 in the United States.

Year	No. of isolates (n)	No. of MLG (g)	g/n	No. and freq. (%) of new MLG	No. and freq. (%) of private MLG	Stoddart and Taylor’s MLGs diversity (G) (CI)^a^	Shannon–Wiener index of MLGs diversity (H) (CI)^b^	*E*_5_^c^	*H*_exp_^d^	rbarD^e^	*p-*value (rbarD)
2010	322	99	0.31	92 (92.9)	90 (90.9)	12.56 (9.039, 16.078)	3.63 (3.44, 3.81)	0.32 (0.26, 0.38)	0.42	0.37	<0.001
2011	319	140	0.44	133 (95.0)	118 (84.3)	17.77 (13.147, 22.402)	4.03 (3.85, 4.20)	0.31 (0.25, 0.36)	0.33	0.33	<0.001
2012	201	160	0.80	152 (95.0)	136 (85.0)	96.42 (81.848, 110.997)	4.91 (4.79, 5.03)	0.71 (0.61, 0.81)	0.41	0.15	<0.001
2013	353	290	0.82	272 (93.8)	248 (85.5)	155.57 (132.42, 178.713)	5.49 (5.39, 5.59)	0.64 (0.54, 0.74)	0.39	0.09	<0.001
2014	315	257	0.82	234 (91.1)	223 (86.8)	146.13 (124.757, 167.511)	5.38 (5.28, 5.48)	0.67 (0.57, 0.77)	0.41	0.11	<0.001
2015	220	177	0.80	154 (87.0)	146 (82.5)	127.37 (115.192, 139.545)	5.05 (4.95, 5.15)	0.82 (0.76, 0.88)	0.42	0.12	<0.001
2016	290	258	0.89	224 (86.8)	219 (84.9)	188.57 (170.076, 207.054)	5.46 (5.37, 5.55)	0.8 (0.72, 0.88)	0.41	0.08	<0.001
2017	227	209	0.92	186 (89.0)	186 (89.0)	186.03 (174.24, 197.81)	5.30 (5.22, 5.39)	0.93 (0.89, 0.97)	0.42	0.07	<0.001
Total	2247	1454	0.65	1447 (99.5)	1366 (93.9)	192.64 (162.61-222.65)	6.71 (6.65, 6.77)	0.23 (0.19, 0.27)	0.43	0.11	<0.001

*^a^Stoddart and Taylor’s Index of MLG diversity and CI (Stoddart and Taylor, 1988) calculated using poppr package in R program 4.3. ^b^Shannon–Wiener Index of MLG diversity and confidence interval (Shannon, 1948) calculated using poppr package in R program 4.3. ^c^Evenness, E_5_ (Grünwald et al., 2003). ^d^Nei’s unbiased gene diversity (H_exp_) (Nei, 1978) over 14 SSR loci. ^e^Standardized index of association, rbarD. p < 0.001 indicates significant disequilibrium due to linkage among loci, indicating populations are clonal (Agapow and Burt, 2001).*

**FIGURE 1:**
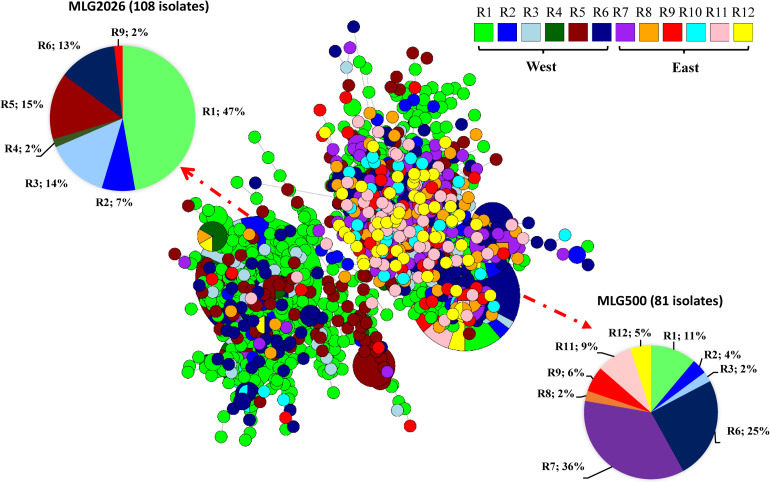
Minimum spanning network representing relationships among multilocus genotypes (MLGs) of *Puccinia striiformis* f. sp. *tritici* identified from 12 epidemiological regions in the United States. Each node represents a different MLG. The size of a node represents the number of isolates in the same MLG. Node colors represent population membership in regions.

The 18 predominant MLGs were not evenly distributed. At the spatial level, MLG1355 and MLG2023 with 12 isolates each were detected only in the west, and the most predominant MLG2026 and MLG1989, MLG1743, MLG2018, MLG1106, and MLG909 were mainly detected in the west regions (R1–R6), with only few isolates detected in the east. The remaining nine predominant MLGs (MLG500, MLG1055, MLG671, MLG1099, MLG1623, MLG1082, MLG1550, MLG27, and MLG650) were almost evenly distributed in the west and east regions (Figure 1 and Supplementary Figure 3). The minimum spanning network showed that the west populations (R1–R6) had more MLGs than the east populations (R7–R12), and a separate branch of MLGs mainly distributed in the west regions. The most predominant MLG (MLG2026) had 98% of its 108 isolates in the west, whereas it had only 2% in the east. The second predominant MLG (MLG500) had 58% in the east and 42% in the west (Supplementary Figure 4).

The frequencies of the 18 predominant MLGs with 10 or more isolates in the 8 years are provided in Supplementary Figure 5. MLG1355, MLG500, and MLG650 were detected only in 2010, and MLG27, MLG671, and MLG2018 only in 2011. The most predominant MLG2026 first appeared in 2010 in a relatively high frequency and further increased in 2011, but gradually decreased from 2012 until it disappeared in 2016. In addition to MLG2026, 10 MLGs (MLG909, MLG989, MLG1082, MLG1099, MLG1055, MLG1550, MLG1623, MLG1989, MLG2023, and MLG1743) were detected in 3 or more years.

### Genotypic and Gene Diversities

The genotypic diversity *G* (Stoddart and Taylor’s index of MLGs diversity) and *H* (Shannon–Wiener index of MLGs diversity) values and their respective 95% confidence intervals by bootstrap statistics for the regional populations are given in Table 2, and the index values and confidence intervals for the yearly populations are given in Table 3. Among the 12 epidemiological regions, R1 had the highest values of *G* (209.13, confidence interval = 166.33–251.93) and *H* (6.29, confidence interval = 6.22–6.36), and the confidence intervals did not overlap with those of the other regions (Table 2). In contrast, R4 had the lowest *G* (12.74, confidence interval = 11.73–13.74) and *H* (2.71, confidence interval = 2.40–3.02) values. Generally, the west regions had relatively high genotypic diversity than the east regions. The *G*-value in the west (across R1–R6) was 162.2 with a confidence interval of 125.52–198.87, which was higher than the *G*-value of 97.6 with a confidence interval of 75.88–119.25 in the east (across R7–R12). A similar result was obtained for the *H*-values, 6.13 (confidence interval = 6.44–6.58) for the west and 5.24 (confidence interval = 5.54–5.76) for the east.

The evenness (*E*_5_) was determined for the distributions of MLGs in the regional population (Table 2). The *E*_5_ values ranged from 0.39 in R1 to 0.83 in R4 in the west with an overall value of 0.24 across all six west regions (R1–R6), whereas the *E*_5_ values ranged from 0.40 in R7 to 0.94 in R10 with an overall value of 0.34 across all six east regions (R7–R12). The expected heterozygosity (*H*_exp_), which represents gene diversity, was estimated for each regional population. The overall heterozygosity across the east region (*H*_exp_ = 0.44) was slightly higher than that of the west region (*H*_exp_ = 0.41). These results indicated that the *Pst* populations in the west were less even, but more diverse than those of the east.

Among the yearly populations, the 2010 and 2011 populations had the lowest *G-* and *H*-values and confidence intervals than those of the other years (Table 3). The 2010 and 2011 populations also had the lowest evenness. Moreover, the 2011 population also had the lowest expected heterozygosity (gene diversity) (*H*_exp_ = 0.33). These results indicated that the 2010 and 2011 populations were less diverse than those of 2012 to 2017.

The AMOVA results revealed significant variations by year and by region (Table 4). Variations among regions, among years within regions, and within years within regions, as well as among years, among regions within years, and within regions within years were all significant (*p* < 0.001). However, the highest variation was within years within regions or within regions within years, indicating that the *Pst* population in a single region of any year was highly diverse.

**TABLE 4:** Analysis of molecular variance for comparing *Puccinia striiformis* f. sp. *tritici* isolates for two different hierarchies: year within region and region within year^a^.

Source of variation	*df*	Sum of squares	Mean squares	Estimated variance	Variation (%)	*p-*value
Region by year						
Among regions	11 (11)	467.09 (224.6)	42.46 (20.4)	0.15 (0.08)	4.91 (2.7)	<0.001 (<0.001)
Among years within regions	80 (79)	1,347.62 (760.1)	16.85 (9.6)	0.62 (0.39)	20.21 (13.1)	<0.001 (<0.001)
Within years within regions	2,155 (1,611)	4,940.85 (4,067.7)	2.29 (2.5)	2.29 (2.5)	74.89 (84.1)	<0.001 (<0.001)
Year by region						
Among years	7 (7)	853.3 (461.9)	121.9 (66.0)	0.3 (0.2)	10.4 (8.0)	<0.001 (<0.001)
Among regions within years	84 (83)	959 (522.8)	11.4 (6.3)	0.5 (0.2)	14.8 (8.0)	<0.001 (<0.001)
Within regions within years	2,155 (1,611)	4,917.9 (4,067.7)	2.3 (2.5)	2.3 (2.5)	74.8 (83.9)	<0.001 (<0.001)

*^a^Clone-corrected values are provided in parentheses. p-values are based on 999 permutations. df, degrees of freedom.*

### Spatial Population Differentiation and Genetic Relationships

**FIGURE 2:**
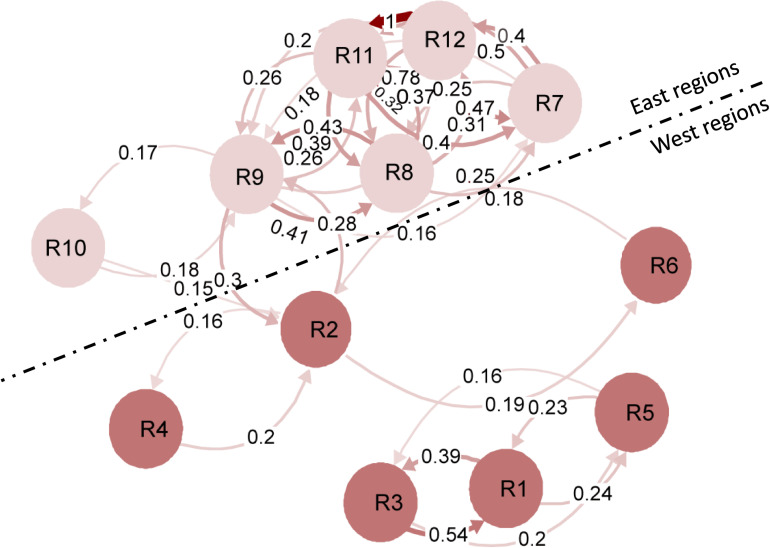
Simplified migration networks to visualize the gene flow patterns of *Puccinia striiformis* f. sp. *tritici* among the 12 regional populations in the United States. Regions are represented by the nodes, and the gene flow strength is represented by the line thickness. The filter threshold was set to 0.15.

The gene differentiation *Gst* values of the 12 regions are shown in Supplementary Figure 6. The *Gst* values (0.005–0.129) were generally high between the west and east populations, but low (0.001–0.051) between the west populations (R1–R6) and even lower (−0.003 to 0.043) between east populations (R7–R12). A migration network was generated based on the *G*st values, showing higher migration rates (lower differentiation) between the east populations than between the west populations (Figure 2). The gene flow (*N*_m_) among the west populations was 17.25, but much higher among the east populations (*N*_m_ = 28.41), indicating that the west populations were more diverse and differentiated than the east populations.

**FIGURE 3:**
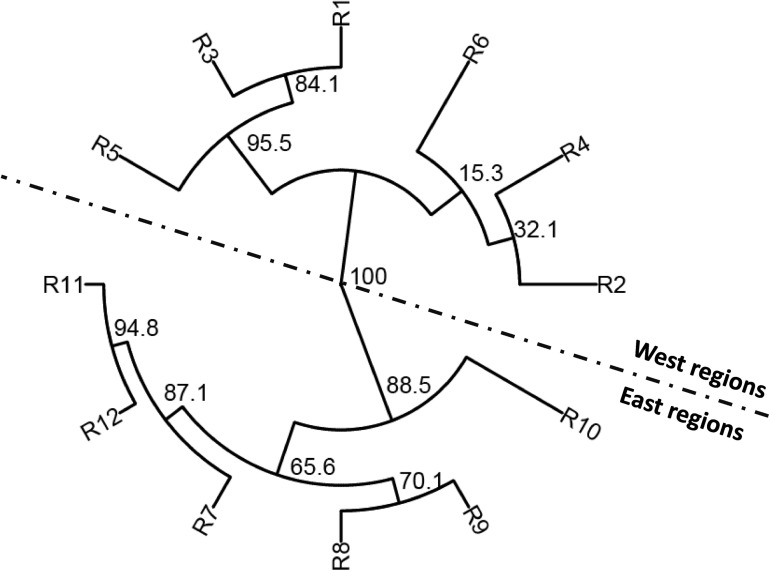
Phylogenetic tree based on Nei’s genetic distances among 12 epidemiological regions of wheat stripe rust in the United States.

To determine the genetic relationships among the populations of different regions, the phylogenetic analysis was conducted using the MLGs based on Nei’s genetic distance (Figure 3). Among the 12 regional populations, the west populations (R1–R6) were clearly separated from the west populations (R7–R12). Among the west populations, R1, R3, and R5 were more closely related, whereas R2, R4, and R6 were more closely related. Among the east populations, R7, R11, and R12 were more closely related, whereas R8, R9, and R10 were more closely related.

### Temporal Population Differentiation and Genetic Relationships

Similarly, the gene differentiation (*Gst*) values of the eight yearly population were calculated and are presented in Table 5. The pairwise differentiations were all significant (*p* < 0.001), indicating that the *Pst* population changed from year to year. The *Gst* values ranged from 0.014 between the 2015 and 2017 populations to 0.223 between the 2011 and 2016 populations. The 2010 and 2011 populations were more differentiated from the other yearly populations. In subsequent years, the differentiation was the highest between the 2010 and 2011 populations (0.154), followed by between 2011 and 2012, and the lowest between 2016 and 2017, indicating a trend of differentiation reduction from 2010 to 2017.

**TABLE 5:** Pairwise *Gst* values among eight yearly populations of *Puccinia striiformis* f. sp. *tritici* in the United States from 2010 to 2017^a^.

Year	2010	2011	2012	2013	2014	2015	2016	2017
2010		<0.001	<0.001	<0.001	<0.001	<0.001	<0.001	<0.001
2011	0.154		<0.001	<0.001	<0.001	<0.001	<0.001	<0.001
2012	0.127	0.110		<0.001	<0.001	<0.001	<0.001	<0.001
2013	0.189	0.184	0.045		<0.001	<0.001	<0.001	<0.001
2014	0.137	0.071	0.029	0.045		<0.001	<0.001	<0.001
2015	0.145	0.146	0.021	0.019	0.023		<0.001	<0.001
2016	0.188	0.223	0.058	0.016	0.060	0.019		<0.001
2017	0.170	0.195	0.035	0.015	0.049	0.014	0.018	

*^a^Gst (Hed) values below the diagonal. p-values based on 999 permutations are shown above the diagonal.*

**FIGURE 4:**
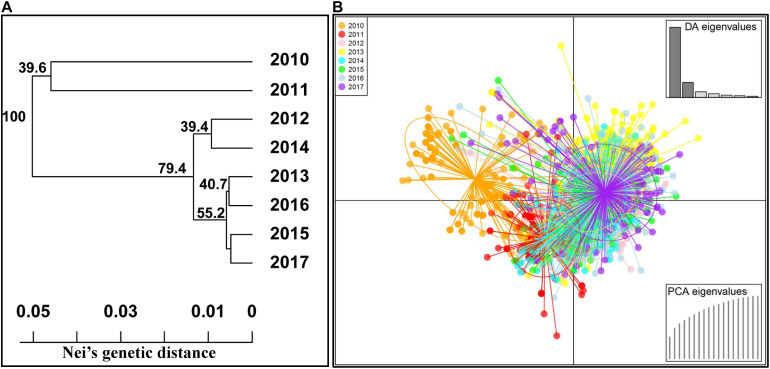
Phylogenetic tree based on Nei’s genetic distances among 8 years **(A)** and discriminant analysis of principal components (DAPC) analysis of all *Puccinia striiformis* f. sp. *tritici* isolates across eight yearly populations **(B)**.

To confirm the genetic relationships among the populations of different years, the phylogenetic analysis was also conducted using the MLGs based on Nei’s genetic distance values (Figure 4A). Among the eight yearly populations, the 2010 and 2011 populations were clearly separated from all other yearly populations. The 2012 population was more related to the 2014 population and similar for the 2013 and 2016 populations and the 2015 and 2017 populations. The 2012 to 2017 populations were more related to each other than to either the 2010 or 2011 population. Moreover, the DAPC analysis based on the predefined yearly populations was also conducted to detect the yearly population relationships (Figure 4B). The results were consistent with the phylogenetic results, revealing the high divergence of the 2010 and 2011 populations from those of the other years.

### Identification of MGs

**FIGURE 5:**
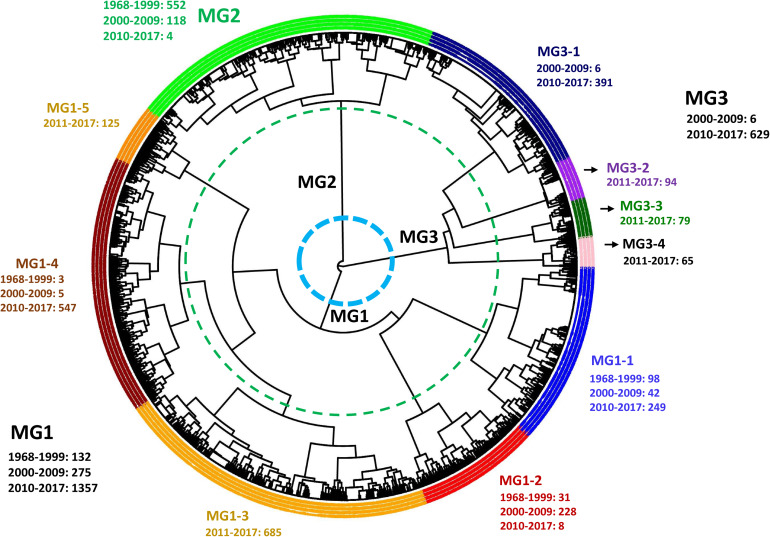
Dendrogram of *Puccinia striiformis* f. sp. *tritici* populations from 1968 to 2009 and 2010 to 2017 in the United States constructed based on dissimilarities assessed with 12 simple sequence repeat markers (SSR) using the hierarchical cluster analysis, showing three major molecular groups (MGs) and 10 sub-MGs and the isolate numbers in 1968 to 2009 and 2010 to 2017.

Hierarchical cluster analysis showed that the 3,330 isolates, including 2,247 isolates of 2010–2017 in the present study and 1,083 isolates of 1968–2009 in the previous study, were optimally grouped into three clusters or MGs, MG1, MG2, and MG3 (Figure 5), of which MG2 was the same as in the previous study, mainly including the isolates of 1968–2009 (Liu et al., 2021), whereas MG1 and MG3 mainly contained isolates of 2010–2017. MG1 was further separated into five sub-MGs (MG1-1, MG1-2, MG1-3, MG1-4, and MG1-5) and MG3 into four sub-MGs (MG3-1, MG3-2, MG3-3, and MG3-4) (Figure 5). The numbers of isolates from 1968–1999, 2000–2009, and/or 2010 to 2017 in each MG and each sub-MG are given in Figure 5. MG1-1 had the highest number of isolates (249) from 2010 to 2017, followed by 1968–1999 (98) and 2000–2009 (42). MG1-2 consisted of 31 isolates from 1968 to 1999, 228 from 2000 to 2009, and 8 from 2010 to 2017. MG1-3 had 685 isolates all from 2010 to 2017. MG1-4 had the most isolates from 2010 to 2017 (547) and only few isolates from 1968 to 1999 (3) and 2000–2009 (5). MG1-5 had all 125 isolates from 2010 to 2017. MG2 had majority isolates (552) from 1968 to 1999, 118 isolates from 2000 to 2009, and only 4 from 2010 to 2017. MG3-1 had majority isolates (391) from 2010 to 2017 and only six from 2000 to 2009, whereas all isolates of MG3-2 (94), MG3-3 (79), and MG3-4 (65) were from 2010 to 2017. These data showed that MG1-3, MG1-5, MG3-2, MG3-3, and MG3-4 emerged and developed during 2010–2017, and the entire MG3 appeared and developed since 2000.

To identify the marker patterns for the 10 sub-MGs, one isolate was selected based on MLG and first year of detection to represent each subgroup (Table 6). MG1-1, MG1-3, MG1-4, and MG1-5 that contained isolates mainly from 2010 to 2017 were heterozygous (H) at a half or more of marker loci. MG3-1, MG3-2, MG3-3, and MG3-4 that also mainly contained the isolates from 2010 to 2017 were homozygous for the major alleles (A) at a half or more loci. MG1-2 differed from other sub-MGs by its homozygous with the minor allele (B) at the RJ21 locus and possible at the RJ8N locus. MG2 differed from other MGs mainly by its homozygosity at the CPS13 locus (Table 6 and Supplementary Table 5).

**TABLE 6:** Allelic genotypes at each simple sequence repeat (SSR) locus of the represent isolate in each molecular group of *Puccinia striiformis* f. sp. *tritici*.

			Pstp002	RJ8N	RJ21	CPS13	Pstp029	CPS04	CPS02	RJ20	CPS08	RJ18	Pstp006	Pstp003
	
MG	MLG	Represent isolate	*A* = 367 *B* = 361	*A* = 333 *B* = 330	*A* = 196 *B* = 205/202	*A* = 149 *B* = 152	*A* = 195 *B* = 198	*A* = 276 *B* = 278/273	*A* = 125 *B* = 127/129	*A* = 311 *B* = 314	*A* = 228 *B* = 213/225/231	*A* = 364 *B* = 358	*A* = 243 *B* = 246	*A* = 226 *B* = 242
MG1-1	MLG500	10–301	H^a^	H	H	H	H	B	B	B	B	H	H	H
MG1-2	MLG67	2004–195	H	B	B	A	A	A	A	H	A	A	B	B
MG1-3	MLG1099	12–26	H	H	H	H	A	H	H	H	A	A	H	H
MG1-4	MLG1642	13–454	H	H	A	H	A	H	A	H	A	A	A	H
MG1-5	MLG1295	15–42	H	A	H	A	H	A	H	H	A	A	A	H
MG2	MLG188	1970–035	A	B	A	B	H	H	H	A	A	B	A	H
MG3-1	MLG2026	10–161	A	A	A	A	A	A	A	A	A	B	B	B
MG3-2	MLG1743	11–175	A	A	A	A	A	A	A	H	B	B	B	B
MG3-3	MLG1780	11–61	A	A	A	A	A	A	A	A	B	B	B	H
MG3-4	MLG833	11–66	A	A	A	A	A	H	H	H	A	B	B	H

*^a^H denotes heterozygotes with both alleles A and B.*

**FIGURE 6:**
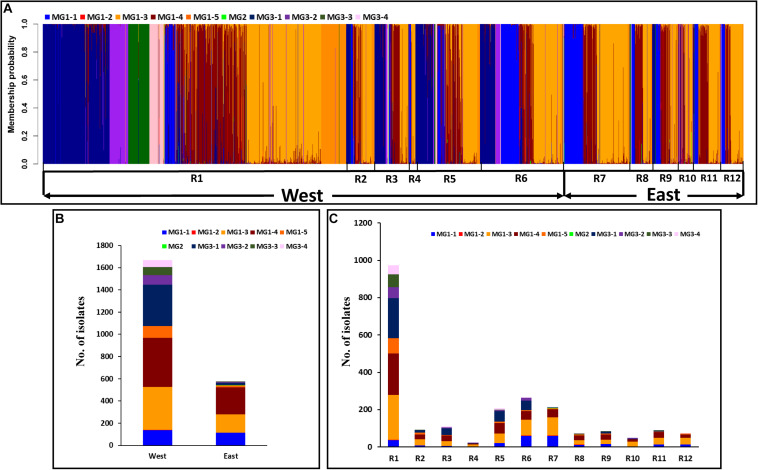
Membership probability of each *Puccinia striiformis* f. sp. *tritici* (*Pst*) isolate in the 10 submolecular groups (MGs) from discriminant analysis of principal components (DAPC) analysis. *Pst* isolates are organized by epidemiological regions given at the bottom **(A)**. Histogram of the number of isolates clustered in the 10 molecular groups from West and East populations **(B)**. Distribution of the number of isolates clustered in the 10 molecular groups across 12 epidemiological regions (R1–R12) in the United States **(C)**.

**FIGURE 7:**
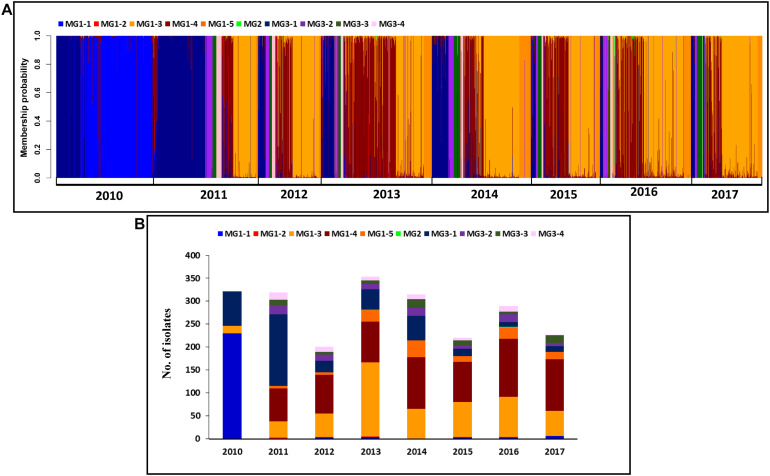
Membership probability for each *Puccinia striiformis* f. sp. *tritici* (*Pst*) isolate in each submolecular group (MG) from discriminant analysis of principal components (DAPC) analysis. *Pst* isolates are organized by 8 years given at the bottom **(A)**. Histogram of the numbers of isolates clustered in the 10 MGs from the 8 years **(B)**.

To further determine the spatial and temporal distributions of MGs and sub-MGs across the epidemiological regions and years, scatterplots and histograms were generated from the DAPC analysis (Figures 6A–C, 7A,B and Supplementary Figures 7, 8). All three MGs were detected in the west regions with 1,073 isolates (64.4%) in MG1 (MG1-1, MG1-3, MG1-4, and MG1-5), 594 isolates (35.6%) in MG3 (mainly in MG3-1, MG3-2, MG3-3, and MG3-4), and only 2 isolates in MG2, whereas most of the isolates (541 isolates, 93.3%) from the east regions were in MG1 (mainly in MG1-1, MG1-3, and MG1-4), 39 isolates (6.7%) in MG3 (mainly in MG3-1), and only 2 isolates in MG2 (Figure 6 and Table 7). Of the 2,247 isolates from 2010 to 2017, 1,614 (71.8%) isolates were clustered into MG1, 633 (28.2%) into MG3, and only 4 (0.2%) in MG2 (Table 7).

**TABLE 7:** Numbers of *Puccinia striiformis* f. sp. *tritici* isolates in the molecular groups (MGs) identified using hierarchical cluster analysis across the 12 epidemiological regions in the United States.

	Number of isolates														
		West							East						
MG	Total	R1	R2	R3	R4	R5	R6	Subtotal	R7	R8	R9	R10	R11	R12	Subtotal
MG1-1	249	37	8	5	4	22	60	136	60	11	15	2	13	12	113
MG1-2	8	3	1	0	0	0	1	5	1	1	1	0	0	0	3
MG1-3	685	239	33	26	10	49	85	442	99	24	22	26	35	37	243
MG1-4	547	223	25	30	4	57	45	384	43	25	30	14	32	19	163
MG1-5	125	80	9	2	0	8	7	106	5	5	3	0	3	3	19
Subtotal	1,614	582	76	63	18	136	198	1,073	208	66	71	42	83	71	541
MG2	4	1	0	1	0	0	0	2	2	0	0	0	0	0	2
MG3-1	391	214	14	36	3	56	49	372	2	2	10	1	3	1	19
MG3-2	94	60	0	5	1	4	16	86	1	0	0	5	1	1	8
MG3-3	79	68	1	0	0	4	0	73	0	3	2	0	1	0	6
MG3-4	65	48	0	4	0	7	2	61	1	2	0	0	0	1	4
Subtotal	633	391	15	46	4	71	67	594	6	7	12	6	5	3	39
Total	2,247	973	91	109	22	207	265	1,667	214	73	83	48	88	74	580

When all isolates were analyzed at the temporal level, 246 isolates (76.4%) from 2010 were in MG1, of which 229 isolates (93.1%) were in MG1-1, and 76 isolates (23.6%) were in MG3. Most isolates (63.6%) from 2011 were in MG3, and 36.4% of isolates were in MG1. Most isolates (68.3–83.4%) from 2012 to 2017 were in MG1, and most remaining isolates (15.8–31.8%) were in MG3 (Figure 7 and Supplementary Figure 8). For the sub-MGs, all 76 isolates from 2010 in MG3 were in MG3-1, and the isolates from 2010 in MG1 were mostly in MG1-1 (93.1%), MG1-4 (6.5%), and MG1-2 (0.4%). MG1-3, MG1-5, MG3-2, MG3-3, and MG3-4 contained isolates only from 2011 to 2017 (Table 8).

**TABLE 8:** Numbers of *Puccinia striiformis* f. sp. *tritici* isolates in the molecular groups (MGs) identified by hierarchical cluster analysis among 8 years from 2020 to 2017.

	Number of isolates								
MG	2010	2011	2012	2013	2014	2015	2016	2017	Subtotal
MG1-1	229	1	3	4	0	3	3	6	249
MG1-2	1	2	1	1	1	1	1	0	8
MG1-3	0	72	84	89	113	88	126	113	685
MG1-4	16	35	51	162	64	76	88	55	547
MG1-5	0	6	6	25	37	12	24	15	125
Subtotal	246	116	145	281	215	180	242	189	1,614
MG2	0	0	0	1	0	0	2	1	4
MG3-1	76	155	25	44	53	16	10	12	391
MG3-2	0	20	14	12	17	7	17	7	94
MG3-3	0	12	6	7	20	11	6	17	79
MG3-4	0	16	11	8	10	6	13	1	65
Subtotal	76	203	56	71	100	40	46	37	629
Total	322	319	201	353	315	220	290	227	2,247

### Mode of Reproduction

The standard IA (rbarD) values ranged from 0.06 in R12 to 0.19 in R3 (Table 2) and 0.07 in 2017 to 0.37 in 2010 (Table 3), with the mean of 0.1 for both the west and east populations and a value of 0.107 for the overall population. All populations had *p* < 0.001 and located outside the bell curve of the simulated distribution (expected from unlinked loci) of a randomly mating population for the *Pst* population (Supplementary Figure 9). Thus, the data rejected the hypothesis of no linkage among markers but supported the clonal mode of reproduction in the *Pst* population.

### Correlation Between the Molecular and Virulence Data

Of the 2,247 isolates genotyped with the SSR markers, 2,221 were tested for virulence phenotypes (Supplementary Table 6). Races in the 18 predominant MLGs associated with multiple races and 50 MLGs each associated with only one race, together with years detected, are listed in Table 9. The major races associated with the 18 predominant MLGs and their distributions in the epidemiological regions and across the years of 2010–2017 are also shown in Supplementary Figures 3, 5. Correlation analysis was conducted using the phenotypic data and SSR marker data. The overall correlation coefficient (*r*), when all of the 2,221 isolates were considered without any grouping, was 0.325 (*p* < 0.001) between the marker genotypes and virulence phenotypes. There were 50 MLGs with more than two isolates each identified as a single race, of which 17 were race PSTv-37 and 11 were race PSTv-52, indicating that many isolates from different regions in different years were identified as the same MLGs or races (Table 9).

**TABLE 9:** Races in major multilocus genotypes (MLGs) of *Puccinia striiformis* f. sp. *tritici* and number of isolates, number of years, and first and last year of detection for each of the MLGs.

MLG^a^	MG^b^	SubMG^*c*^	No. of races	Race (No. of isolates)^d^	No. of isolates	No. of years	First year	Last year
MLG2026	MG3	MG3-1	9	PSTv-11(74), PSTv-14(23), PSTv-4(2), PSTv-17(3), PSTv-37(1), PSTv-39(1), PSTv-40(1), PSTv-49(2), PSTv-175(1)	108	6	2010	2016
MLG500	MG1	MG1-1	15	PSTv-37(33), PSTv-35(8), PSTv-36(8), PSTv-11(1), PSTv-14(1), PSTv-30(3), PSTv-32(2), PSTv-33(2), PSTv-34(3), PSTv-41(2), PSTv-284(1), PSTv-16(1), NT(2), PSTv-31(1), PSTv-101(2), PSTv-317(1)	81	1	2010	2010
MLG1055	MG1	MG1-3	6	PSTv-37(7), PSTv-52(12), PSTv-198(5), PSTv-41(1), PSTv-19(1), PSTv-101(1)	27	4	2012	2016
MLG671	MG1	MG1-3	5	PSTv-37(14), PSTv-31(6), PSTv-34(4), PSTv-30(1), PSTv-35(1)	26	1	2011	2011
MLG1099	MG1	MG1-3	6	PSTv-37(10), PSTv-52(10), PSTv-73(1), PSTv-75(1), PSTv-78(1), PSTv-198(1)	24	6	2012	2017
MLG1743	MG3	MG3-2	9	PSTv-4(7), PSTv-53(6), PSTv-11(2), PSTv-17(1), PSTv-28(1), PSTv-39(1), PSTv-77(1), PSTv-120(1), PSTv-212(2)	22	7	2011	2017
MLG1989	MG3	MG3-1	5	PSTv-11(12), PSTv-14(2), PSTv-4(1), PSTv-17(1), PSTv-49(1)	17	3	2010	2014
MLG1623	MG1	MG1-4	5	PSTv-37(4), PSTv-52(9), PSTv-41(1), PSTv-53(1), PSTv-198(1)	16	4	2012	2016
MLG2018	MG3	MG3-1	3	PSTv-11(13), PSTv-14(1), PSTv-37(1)	15	1	2011	2011
MLG1082	MG1	MG1-3	2	PSTv-37(9), PSTv-52(4)	13	4	2012	2017
MLG1550	MG1	MG1-4	5	PSTv-37(2), PSTv-52(8), PSTv-14(1), PSTv-43(1), PSTv-120(1)	13	3	2013	2015
MLG1355	MG1	MG1-1	3	PSTv-18(5), PSTv-19(6), PSTv-65(1)	12	1	2010	2010
MLG27	MG1	MG1-1	4	PSTv-37(7), PSTv-35(2), PSTv-30(1), PSTv-36(1)	12	1	2010	2010
MLG2023	MG3	MG3-1	5	PSTv-4(6), PSTv-53(2), PSTv-11(1), PSTv-120(2), PSTv-15(1)	12	6	2011	2017
MLG650	MG1	MG1-3	6	PSTv-37(4), PSTv-30(2), PSTv-31(3), PSTv-35(1), PSTv-34(1), PSTv-40(1)	12	1	2011	2011
MLG1106	MG1	MG1-3	4	PSTv-52(8), PSTv-11(1), PSTv-37(1), PSTv-62(1)	11	2	2014	2015
MLG989	MG1	MG1-3	4	PSTv-37(5), PSTv-52(4), PSTv-14(1), PSTv-41(1)	11	4	2011	2015
MLG909	MG1	MG1-3	5	PSTv-37(4), PSTv-52(3), PSTv-41(1), PSTv-76(1), PSTv-78(1)	10	3	2011	2015
MLG730	MG1	MG1-1	1	PSTv-12(2)	2	1	2010	2010
MLG747	MG1	MG1-1	1	PSTv-68(2)	2	1	2010	2010
MLG797	MG1	MG1-3	1	PSTv-37(2)	2	1	2017	2017
MLG818	MG3	MG3-4	1	PSTv-48(2)	2	1	2016	2016
MLG861	MG1	MG1-3	1	PSTv-37(2)	2	1	2017	2017
MLG879	MG1	MG1-3	1	PSTv-120(2)	2	1	2017	2017
MLG899	MG1	MG1-3	1	PSTv-37(2)	2	1	2012	2012
MLG1059	MG1	MG1-3	1	PSTv-52(2)	2	1	2013	2013
MLG1073	MG1	MG1-3	1	PSTv-37(2)	2	1	2017	2017
MLG1086	MG1	MG1-3	1	PSTv-37(2)	2	1	2015	2015
MLG1117	MG1	MG1-3	1	PSTv-52(2)	2	1	2014	2014
MLG1164	MG1	MG1-4	1	PSTv-48(2)	2	1	2012	2012
MLG1272	MG1	MG1-3	1	PSTv-37(2)	2	1	2012	2012
MLG1274	MG1	MG1-3	1	PSTv-37(2)	2	1	2012	2012
MLG1312	MG1	MG1-4	1	PSTv-48(2)	2	1	2015	2015
MLG1341	MG3	MG3-1	1	PSTv-11(2)	2	1	2010	2010
MLG1346	MG1	MG1-4	1	PSTv-41(2)	2	1	2010	2010
MLG1509	MG1	MG1-4	1	PSTv-37(2)	2	1	2011	2011
MLG1515	MG1	MG1-4	1	PSTv-37(2)	2	1	2011	2011
MLG1558	MG1	MG1-4	1	PSTv-52(2)	2	1	2015	2015
MLG1677	MG1	MG1-4	1	PSTv-52(2)	2	1	2015	2015
MLG1697	MG1	MG1-4	1	PSTv-74(2)	2	1	2014	2014
MLG1720	MG3	MG3-2	1	PSTv-4(2)	2	1	2014	2014
MLG1940	MG1	MG1-4	1	PSTv-8(2)	2	1	2010	2010
MLG1970	MG3	MG3-1	1	PSTv-14(2)	2	1	2010	2010
MLG1983	MG1	MG1-4	1	PSTv-14(2)	2	1	2010	2010
MLG2047	MG1	MG1-4	1	PSTv-52(2)	2	1	2013	2013
MLG834	MG3	MG3-4	1	PSTv-48(2)	2	2	2014	2016
MLG862	MG1	MG1-3	1	PSTv-52(2)	2	2	2015	2016
MLG1049	MG1	MG1-3	1	PSTv-52(2)	2	2	2013	2016
MLG1084	MG1	MG1-3	1	PSTv-52(2)	2	2	2013	2016
MLG1088	MG1	MG1-3	1	PSTv-37(2)	2	2	2012	2017
MLG1622	MG1	MG1-5	1	PSTv-52(2)	2	2	2013	2014
MLG1714	MG3	MG3-2	1	PSTv-53(2)	2	2	2013	2016
MLG1740	MG3	MG3-2	1	PSTv-4(2)	2	2	2013	2014
MLG729	MG1	MG1-1	1	PSTv-37(3)	3	1	2010	2010
MLG984	MG1	MG1-3	1	PSTv-37(3)	3	1	2012	2012
MLG1445	MG3	MG3-1	1	PSTv-14(3)	3	1	2010	2010
MLG1606	MG1	MG1-4	1	PSTv-52(3)	3	1	2013	2013
MLG1640	MG1	MG1-4	1	PSTv-37(3)	3	1	2012	2012
MLG1777	MG3	MG3-3	1	PSTv-140(3)	3	1	2015	2015
MLG1827	MG1	MG1-4	1	PSTv-14(3)	3	1	2010	2010
MLG897	MG1	MG1-3	1	PSTv-52(3)	3	2	2013	2015
MLG1730	MG3	MG3-2	1	PSTv-4(3)	3	2	2011	2013
MLG980	MG1	MG1-3	1	PSTv-37(4)	4	1	2012	2012
MLG1971	MG3	MG3-1	1	PSTv-11(4)	4	2	2010	2011
MLG1080	MG1	MG1-3	1	PSTv-37(5)	5	1	2012	2012
MLG1087	MG1	MG1-3	1	PSTv-37(5)	5	1	2012	2012
MLG1149	MG1	MG1-3	1	PSTv-37(5)	5	2	2012	2017
MLG1397	MG3	MG3-1	1	PSTv-11(7)	7	1	2010	2010

*^a^MLGs were determined based on 14 SSR loci using the “mlg.vector” function within the “poppr” package in the R program 4.0.3. ^b^Molecular groups (MGs) were determined based on 12 SSR loci (excluding PstP001 and PstP005) using the “hclust” function within the stats package in the R program 4.0.3. ^c^Submolecular groups were determined based on 12 SSR loci (excluding PstP001 and PstP005) using the “hclust” function within the stats package in the R program 4.0.3. ^d^NT, not tested.*

Interestingly, for all 18 predominant MLGs, more than 60% of isolates were identified as two or three predominant races. Predominant MLGs (MLG2026, MLG1989, and MLG2018) clustered in MG3-1 had 90, 75, and 77% of the isolates, respectively, identified as PSTv-11 and PSTv-14, which are closely related races, and all three MLGs shared common alleles at 12 of the 14 SSR loci, except PstP002 that was different between MLG2026 and MLG1989 and RJ8N that was different between MLG2026 and MLG2018. Two predominant MLGs (MLG500 and MLG27) in MG1 had 60 and 82% of the isolates identified as PSTv-37, PSTv-35, and PSTv-36, also closely related races, and MLG500 shared alleles at all 14 marker loci except for RJ21 with MLG27. Both MLG671 and MLG650 were in MG1-3 and shared the same alleles at all 14 marker loci except the CPS04 locus. The isolates of the two MLGs were identified as the same or similar races with 82% of isolates of MLG671 as PSTv-37, PSTv-31, and PSTv-34 and 82% of isolates of MLG650 as PSTv-37, PSTv-31, and PSTv-30. Four MLGs (MLG500, MLG27, MLG650, and MLG671) that had similar races shared all marker loci except CPS02, CPS04, and RJ21, whereas MLG1355 clustered together with MLG500 and MLG27 in MG1 shared only four loci (CPS08, RJ20, Pstp001, and Pstp006) with 60% of isolates identified as old complex races PSTv-18 and PSTv-19. Eight MLGs (MLG1055, MLG1099, MLG1623, MLG1082, MLG1550, MLG989, MLG1106, and MLG909) in MG1 (MG1-4 and MG1-3) had close genetic relationships as they shared 10 of the 14 marker loci (except for CPS02, CPS08, Pstp003, and Pstp006 that were also shared by most of these MLGs with different alleles at only one to four loci). Similarly, these MLGs had 67–100% of their isolates identified as closely related races PSTv-37 and PSTv-52. Moreover, MLG1743 and MLG2023 in MG3 (MG3-2 and MG3-1, respectively), which shared the same alleles at all 14 loci except the CPS08 locus, had 92 and 83% of isolates identified as closely related races PSTv-4 and PSTv-53 (Table 9).

## Discussion

Fourteen SSR markers used in the present study produced adequate number of MLGs in the *Pst* isolates collected throughout the United States from 2010 to 2017. For example, with 13 markers, 97.8% of the MLGs identified with the 14 markers could be identified. Identified MLGs were different in both numbers and genotypes between the west and east regions. More MLGs identified in the west regions than in the east regions were due to the differences in climatic conditions for the survival of the pathogen, the frequent occurrence and development of stripe rust epidemics, number of cultivars, and resistance genes utilized in different regions. Only 85 MLGs (6%) were commonly detected in the west and east regions, indicating few exchanges of inocula between the two big regions. Also, many shared MLGs that mainly distributed in the east were likely from the west, because *Pst* can be more likely spread from west to east than from east to west because of the prevailing wind direction from the west to the east (Chen, 2005). Eight MLGs shared mainly between R6 in the west and R7 in the east demonstrate that inoculum exchanges have occurred between these two regions. Of the 13 MLGs that were detected only in the east regions, 9 were shared by all six east regions (R6–R12), and 4 MLGs were detected only in R7. In contrast, of the 93 MLGs detected only in the west regions, 33 were shared by all six regions (R1–R6), and the other 60 MLGs were detected in only one region, especially R1. This comparison indicates that inoculum exchanges have occurred more frequently among the east regions than among the west regions. This may be related to the lack of natural barriers, relatively uniform crops, and wide growth of the same or similar cultivars in the east regions (Chen et al., 2010; Wan and Chen, 2012; Wan et al., 2016).

In the present study, 6 of the 18 MLGs with 10 or more isolates appeared only in 2010 and/or 2011 and then completely disappeared, indicating that these large MLGs might be totally eradicated or reduced to an undetectable level afterward. The disappearance of these MLGs could be attributed to the changes of wheat cultivars, large-scale application of foliar fungicides, and cold winter and/or dry summer conditions that are unfavorable to the pathogen in some of the years (Chen, 2014, 2020; Wan and Chen, 2016; Wan et al., 2016).

The diversity of the overall population and those of individual epidemiological regions were high, and obvious differences in diversity were observed among the 12 regions (R1–R12) and between the west and east regions. The Stoddart and Taylor’s index values of MLG diversity (*G*), together with the 95% confidence interval, demonstrated that the west populations had a significant higher diversity than the east populations. Especially, R1 (eastern Washington, northeastern Oregon, and northern Idaho), which is one of the most important wheat production regions and the most frequent and severe stripe rust epidemic regions in the United States, had the highest *G*-value. This region also has the highest number of diverse *Pst* races every year (Line and Qayoum, 1992; Chen et al., 2002, [Bibr B20][Bibr B44]; Wan and Chen, 2012, 2014; Wan et al., 2016). The evenness (*E*_5_) was low in this region and also relatively low in the west regions (R1–R6) compared with the east regions (R7–R12) as the MLGs were not evenly distributed, and some MLGs were highly predominate in the west, especially in R1. The high diversity and differentiation in the west regions can be attributed to the high diversity of wheat cultivars with different resistance genes; cropping systems; climatic conditions more favorable to the pathogen survival and infection; and geographic barriers as previously pointed out (Chen et al., 2010; Wan and Chen, 2012, 2014; Wan et al., 2016; Liu et al., 2020; Mu et al., 2020). In contrast, the east regions had overall relatively low diversity as measured by either Stoddart and Taylor’s MLGs diversity and Shannon–Wiener index of MLG diversity, and relatively high evenness and gene flow. The low diversity and differentiation of *Pst* in the east are consistent with the virulence data (Wan and Chen, 2016; Wan et al., 2016; Wan et al., unpublished data). Compared to the west, the relative small number of samples may limit the detection of genotypes at low frequencies in the east regions. However, the vast majority of the east region is flat, and the same or similar wheat cultivars are grown in large acreages, which selects similar races or genotypes adapted to the cultivars and environment. The relatively uniform geographic topology and lack of mountainous barriers allow long-distance dissemination of *Pst* genotypes from relatively few overseason sources.

Although there were some differences among the epidemiological regions in heterozygosity that measures gene diversity, the values of the different regions were all below 0.5, and the overall heterozygosity was low (0.43). The low heterozygosity or gene diversity could be due to the clonal production of *Pst* as discussed below, as inbreeding increases the frequency of homozygotes at the expense of heterozygotes (Allendorf and Utter, 1979). The west population had relatively low heterozygosity (0.41) compared to the east population (0.43). This could be related to the fact that a group of races represented by PSTv-14 was predominant in 2010 and 2011 in the west but seldom detected in the east (Wan and Chen, 2012, 2014; Wan et al., 2016), and the group is more homozygous than the races predominant in the east based on genome sequences (Cuomo et al., 2012). This group of races is represented by MLG-2026 in MG3-1 (Supplementary Figure 3), which is homozygous at all 14 SSR loci (Table 6). In contrast, MLG-27 in MG1-1, corresponding to the race group represented by PSTv-37, is heterozygous at 8 of the 14 SSR loci.

The analysis of the *Pst* populations over the 8 years from 2010 to 2017 allowed us to monitor dynamic changes, and the comparison with the previously identified MLGs from the 1968–2009 collections (Liu et al., 2021) provided a clear picture of dynamics for a much longer period. To track changes of MLGs over the years, all MLGs in the present study were named continuously including those identified from the 1968–2009 collections (Liu et al., 2021). Ideally, the 14 SSR markers used in the previous study (Liu et al., 2021) should have been used in the present study. Unfortunately, 2 (PstP004 and PstP033) of their 14 markers did not amplify any fragments for many isolates in the initial screening and were replaced by PstP001 and PstP005 in the present study, as we attempted to use markers all codominant and no missing loci. As we identified several sub-MGs appeared after 2010 in the present study, and deletion was found in *Pst* isolates for a genomic region carrying avirulence cluster (Xia et al., 2020), we hypothesize that the PstP004 and PstP033 loci could be deleted from some of the recent isolates. Further studies are needed to test the hypothesis for understanding the pathogen evolution. Nevertheless, we named the different MLGs based on marker types at the 12 commonly used markers to be consistent for the MLGs from 1968 to the present. A total of 2,060 MLGs were identified, including 614 MLGs identified from the 1968–2017 collections in the previous study (Liu et al., 2021) and 1,446 new MLGs identified from the 2010–2017 collections in the present study. Eight MLGs (MLG7, MLG117, MLG130, MLG496, MLG499, MLG500, MLG536, and MLG562) were detected in both studies. MLG27 was first detected in 2003 as one isolate (Liu et al., 2021), but was not detected until 2010 when it was detected from 12 isolates in that year. Similarly, MLG500 was first detected in 2004 also as one isolate (Liu et al., 2021) but suddenly detected from 81 isolates identified in 2010. Most isolates (99.4%) of the 1,454 MLGs identified in the present study were not detected in the previous study. Furthermore, 1,436 (98.8%) of the MLGs were detected from <10 isolates in the present study. Both percentages of new MLGs and private MLGs over the years were very high, all greater than 80% (Table 3) in individual years and across the 8 years. The AMOVA revealed that the genetic variation was higher among years than that among regions. All these results indicate that the *Pst* population changes fast.

The clonal reproduction of *Pst* populations in the United States was confirmed based on the test of linkage disequilibrium in the present study. The IA was originally developed as a measure of multilocus linkage disequilibrium (Brown et al., 1980a, b) and was found to be able to detect signatures of sexual reproduction and population structure (Brown et al., 1980a, b; Smith et al., 1993). Based on the values of rbarD, which is a standardization of IA (Agapow and Burt, 2001), the present study did not support sex reproduction for the *Pst* population in the United States as the observed rbarD values were far from the theoretical distribution region of the rbarD values (*p* < 0.001). The result of the present study is consistent with the previous reports of lack of sexual reproduction for the *Pst* population in the United States (Cheng and Chen, 2014; Wang and Chen, 2015; Wang et al., 2015; Liu et al., 2021) and in the world (Bahri et al., 2009b; Ali et al., 2010). Thus, sexual recombination cannot be a major mechanism for the diversity observed in the *Pst* population. Because *Pst* is a dikaryon, clonal reproduction protects the polymorphism at each single locus within isolates, due to the fixed heterozygosity through the “Meselson effect” (Balloux et al., 2003). Much of the genetic variation observed in the present study was maintained in individual isolates.

Mutation should be the major mechanism for explaining the high diversity as most MLGs in the same MG or sub-MGs had different alleles at one or few marker loci, and the stepwise mutation pattern could be commonly found for MLGs in the same groups (Supplementary Table 3). For example, MLG496-1, which was first detected in 2009, differed from MLG130-1, which was detected in 2007, which differed only at the RJ18 locus (changing from 358/364 bp in MLG130-1 to 358/358 in MLG496-1). Using ethyl-methanesulfonate mutagenesis, different genotypes or races can be generated from a single *Pst* isolate (Li et al., 2019b, 2020). Somatic recombination is a plausible mechanism contributing some part of the genetic variation, especially for the formation of new sub-MGs detected in the present study. In our laboratory, somatic recombination has been demonstrated to generate new races or genotypes under controlled conditions (Lei et al., 2017). In *Puccinia graminis* f. sp. *tritici*, the wheat stem rust pathogen, races virulent to resistance gene *Sr50* were found to be produced by somatic recombination that caused loss of *AvrSr50* (Chen et al., 2017), and somatic hybridization caused emergence of the Ug99 race lineage (Li et al., 2019a). As *Pst* migration and incursion from country to country, or from continent to continent, are well known (Wellings and McIntosh, 1990; Steele et al., 2001; Hovmøller et al., 2002, 2008, 2016; Sharma-Poudyal et al., 2013, 2020; Ali et al., 2014), some of the MLGs, especially those in the sub-MGs that suddenly appeared in 2010 and 2011 found in the present study (Figure 5) were likely introduced to the United States.

The correlation coefficient (0.325) between the molecular and virulence data in the present study was comparable with the value of 0.34 reported between the virulence phenotypes and molecular genotype data for the United States *Pst* populations of 2010 and 2011 using secreted protein gene–derived SNP markers (Xia et al., 2016). Based on the MLG relationships with phenotypes, 50 MLGs with more than two isolates from different years and regions were identified as a single race. For the 18 predominant MLGs (Supplementary Figures 3, 5), more than 60% of their isolates were identified as two or three predominant races. For example, MLG2026, MLG1989, and MLG2018 were identified mainly as PSTv-11 and PSTv-14, and they were clustered into MG3-1 and shared 12 homozygous loci out of the 14 loci. The high homozygosity of this race group was consistent with the study by Cheng et al. (2016). Eight MLGs (MLG1055, MLG1099, MLG1623, MLG1082, MLG1550, MLG989, MLG1106, and MLG909) with isolates mainly identified as two closely related races (PSTv-37 and PSTv-52) were clustered into MG1 (MG1-4 and MG1-3) and shared alleles at 10 of 14 loci. Therefore, races PSTv-37 and PSTv-52, which have been predominant throughout the United States in the last decade (Wan and Chen, 2014; Wan et al., 2016; Liu et al., 2017, 2021), are genetically similar. The results show that, although isolates in the same MLG can be different races and *vice versa*, correspondence existed between molecular characterization and virulence phenotypes. Thus, it is possible to quickly identify isolates into race groups using molecular markers for providing information that can be immediately used for managing stripe rust based on resistance gene in growing wheat cultivars.

## Data Availability Statement

The raw data supporting the conclusions of this article will be made available by the authors, without undue reservation.

## Author Contributions

XC, AW, and MW collected stripe rust samples. QB, AW, and MW participated in spore multiplication and DNA extraction, and SSR marker genotyping. QB was a major contributor in DNA extraction, marker genotyping, analyzed and interpreted the data, and in writing the manuscript. DS provided equipment for genotyping, guided data collection, and revised manuscript. XC conceived the study, coordinated stripe rust sample collection, designed the experiments, and wrote the manuscript. All authors reviewed and approved the final manuscript.

## Disclaimer

Mention of trade names or commercial products in this publication is solely for the purpose of providing specific information and does not imply recommendation or endorsement by the United States Department of Agriculture. USDA is an equal opportunity provider and employer.

## Conflict of Interest

The authors declare that the research was conducted in the absence of any commercial or financial relationships that could be construed as a potential conflict of interest.

## Footnotes

1 https://striperust.wsu.edu

2 https://striperust.wsu.edu/races/data/

## Supplementary Material

Supplementary Figure 1 Epidemiological regions in the United States and numbers of stripe rust samples of wheat, caused by *Puccinia striiformis* f. sp. *tritici*, collected from 2010 to 2017.

Supplementary Figure 2 Number of multilocus genotypes (MLGs) detected as a function of the number of loci resampled within the 14 simple sequence repeat (SSR) markers. The box represents the average, minimum, and maximum numbers of MLGs detected when resampling on loci.

Supplementary Figure 3 The multilocus genotypes (MLGs) with 10 or more isolates of *Puccinia striiformis* f. sp. *tritici* and their relationships to races and distributions in 12 epidemical regions **(A)** and the West and East populations **(B)** of the United States.

Supplementary Figure 4 Minimum spanning network representing relationships among multilocus genotypes (MLGs) identified from the west and east populations of *Puccinia striiformis* f. sp. *tritici* in the United States. Each node represents a different MLG. The size of a node represents the number of isolates in the same MLG. Node colors represent memberships of the west (green) and east (blue) populations.

Supplementary Figure 5 Multilocus genotypes (MLGs) with a large number of isolates (≥10) of *Puccinia striiformis* f. sp. *tritici* and their distribution in each of the 8 years (2010–2017) in the United States. Race number, main races, and percentages of main races in respective MLG are behind each bar. The MLGs that appeared in only 1 year was labeled by a star.

Supplementary Figure 6 Values of gene differentiation (*Gst*, Hedrick) among the 12 regional populations of *Puccinia striiformis* f. sp. *tritici* in the United States.

Supplementary Figure 7 **(A)** Membership probability of each isolate of *Puccinia striiformis* f. sp. *tritici* (*Pst*) in the three major molecular groups from discriminant analysis of principal components (DAPC) analysis. *Pst* isolates are organized by epidemiological regions given at the bottom. **(B)** Histogram of the numbers of isolates clustered in the three major molecular groups (MGs) from the West and East populations. **(C)** Distribution of the numbers of isolates clustered in the three major MGs across 12 epidemiological regions (R1–R12) in the United States.

Supplementary Figure 8 **(A)** Membership probability for each isolate of *Puccinia striiformis* f. sp. *tritici* (*Pst*) in three major molecular groups (MGs) from discriminant analysis of principal components (DAPC) analysis. *Pst* isolates are organized by 8 years given at the bottom. **(B)** Histogram showing the numbers of isolates in the three major MGs from 2010–2017 in the United States.

Supplementary Figure 9 Histograms of values of rbrD calculated from 999 permutations. The observed values of rbarD are represented as vertical dashed lines outside the distributions. The hypothesis of no linkage among markers is rejected, indicating a clonal population.

Supplementary Table 1 Sequences, repeat motifs, and annealing temperatures of 14 simple sequence repeat (SSR) primers, used for genetic characterization of *Puccinia striiformis* f. sp. *tritici* isolates collected from 2010 to 2017 in the United States.

Supplementary Table 2 Alleles of 14 SSR loci in 2,247 *Puccinia striiformis* f. sp. *tritici* isolates collected from 2010 to 2017 in the United States.

Supplementary Table 3 Multi-locus genotypes of 14 SSR loci in 2,247 *Puccinia striiformis* f. sp. *tritici* isolates collected from 2010 to 2017 in the United States.

Supplementary Table 4 The Multi-locus genotypes (MLG) shared by different epidemiological regions of *Puccinia Striiformis* f. sp. *tritici* in United States.

Supplementary Table 5 Alleles at the 12 SSR loci with the typical isolate of each MLG in each molecular group.

Supplementary Table 6 Summary of *Puccinia striiformis* f. sp. *tritici (Pst)* isolates from isolates collected from the United States in 2010 to 2017.
