# Supplementary figures and images for: Population Diversity, Dynamics, and Differentiation of Wheat Stripe Rust Pathogen Puccinia striiformis f. sp. tritici From 2010 to 2017 and Comparison With 1968 to 2009 in the United States
Source: Front Microbiol. 2021 Jul 22;12:696835. doi: 10.3389/fmicb.2021.696835 (PMC8339480; doi:10.3389/fmicb.2021.696835)

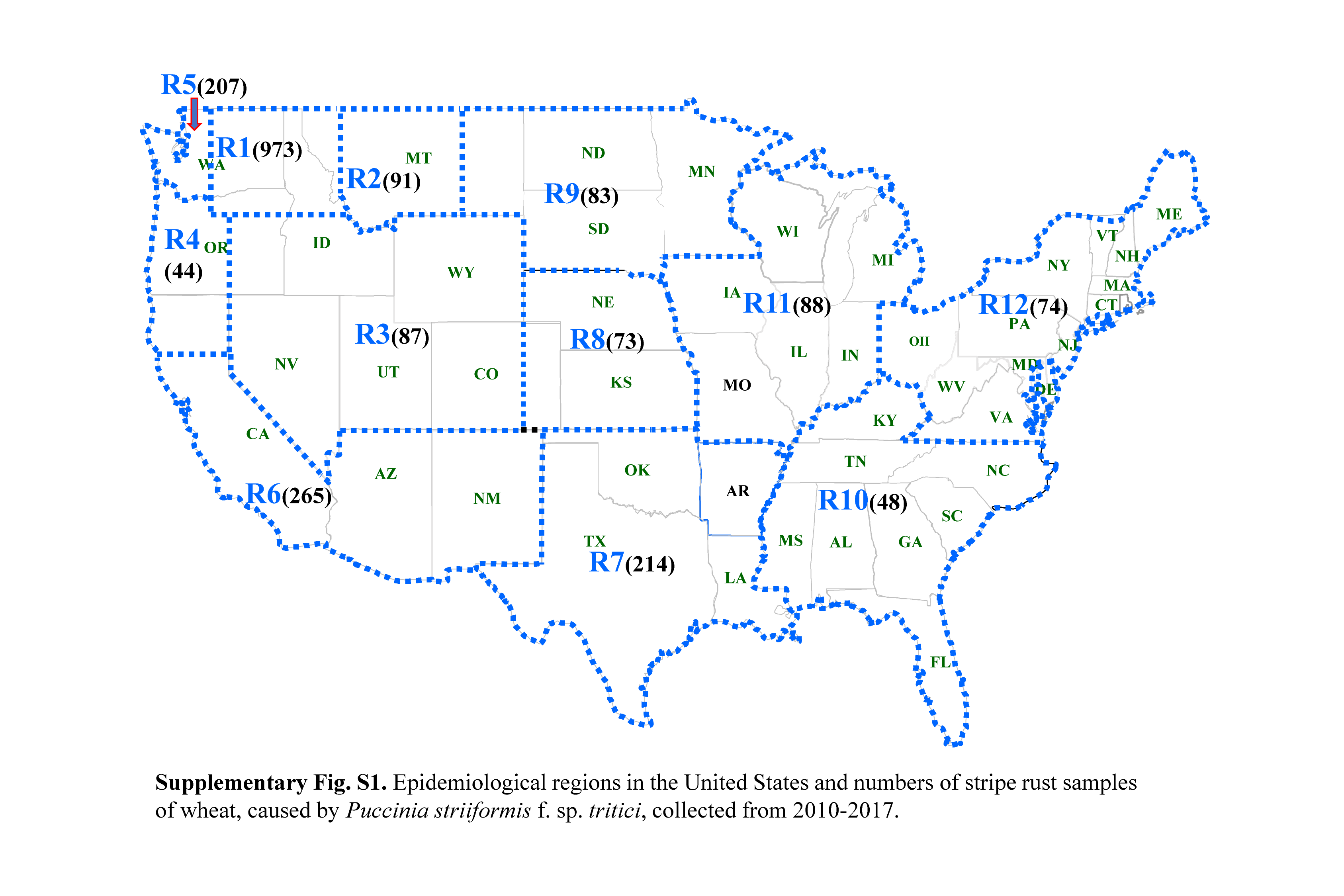

Supplement: Supplementary Figure 1 — Epidemiological regions in the United States and numbers of stripe rust samples of wheat, caused by Puccinia striiformis f. sp. tritici, collected from 2010 to 2017. [file Image_1.TIF]

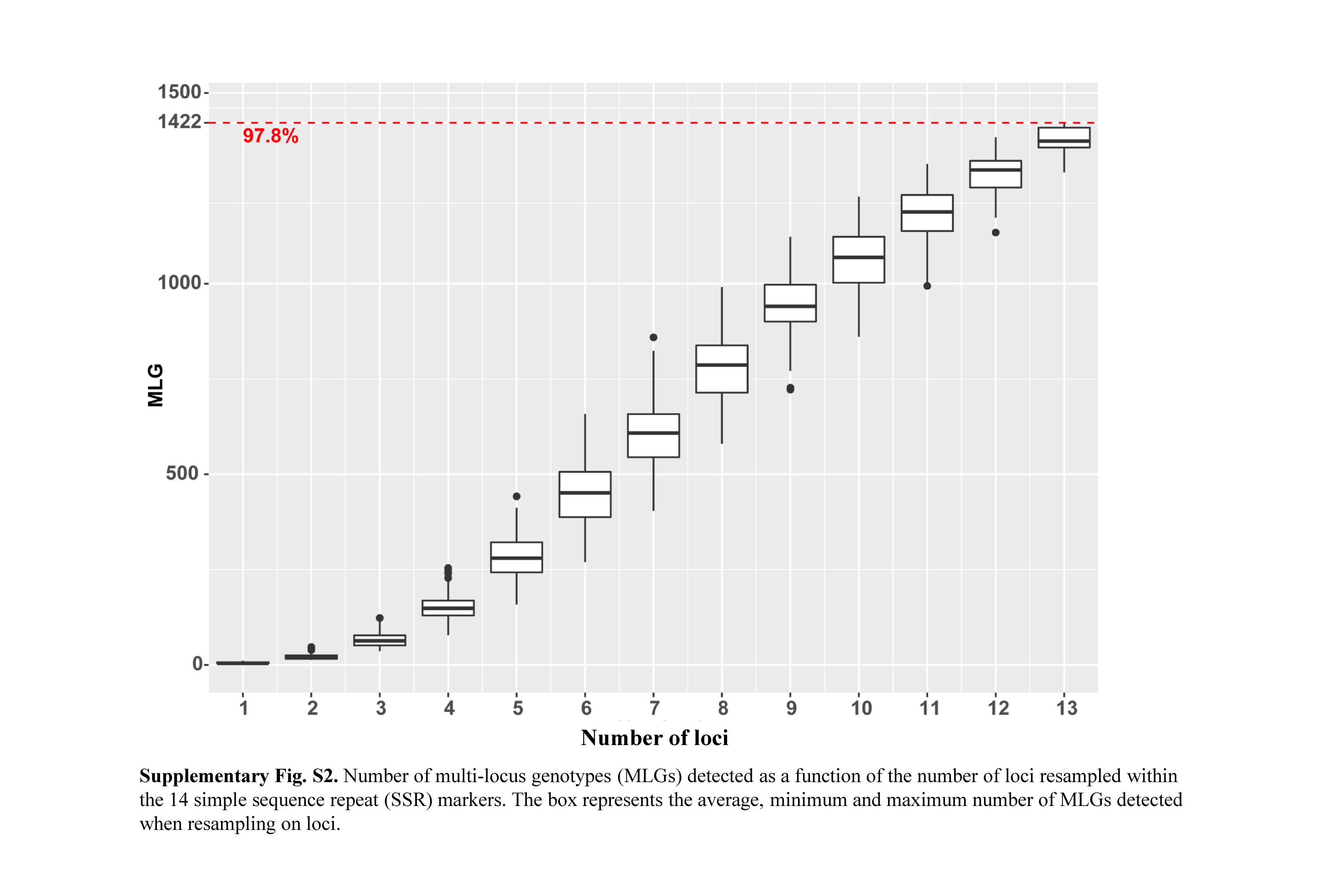

Supplement: Supplementary Figure 2 — Number of multilocus genotypes (MLGs) detected as a function of the number of loci resampled within the 14 simple sequence repeat (SSR) markers. The box represents the average, minimum, and maximum numbers of MLGs detected when resampling on loci. [file Image_2.TIF]

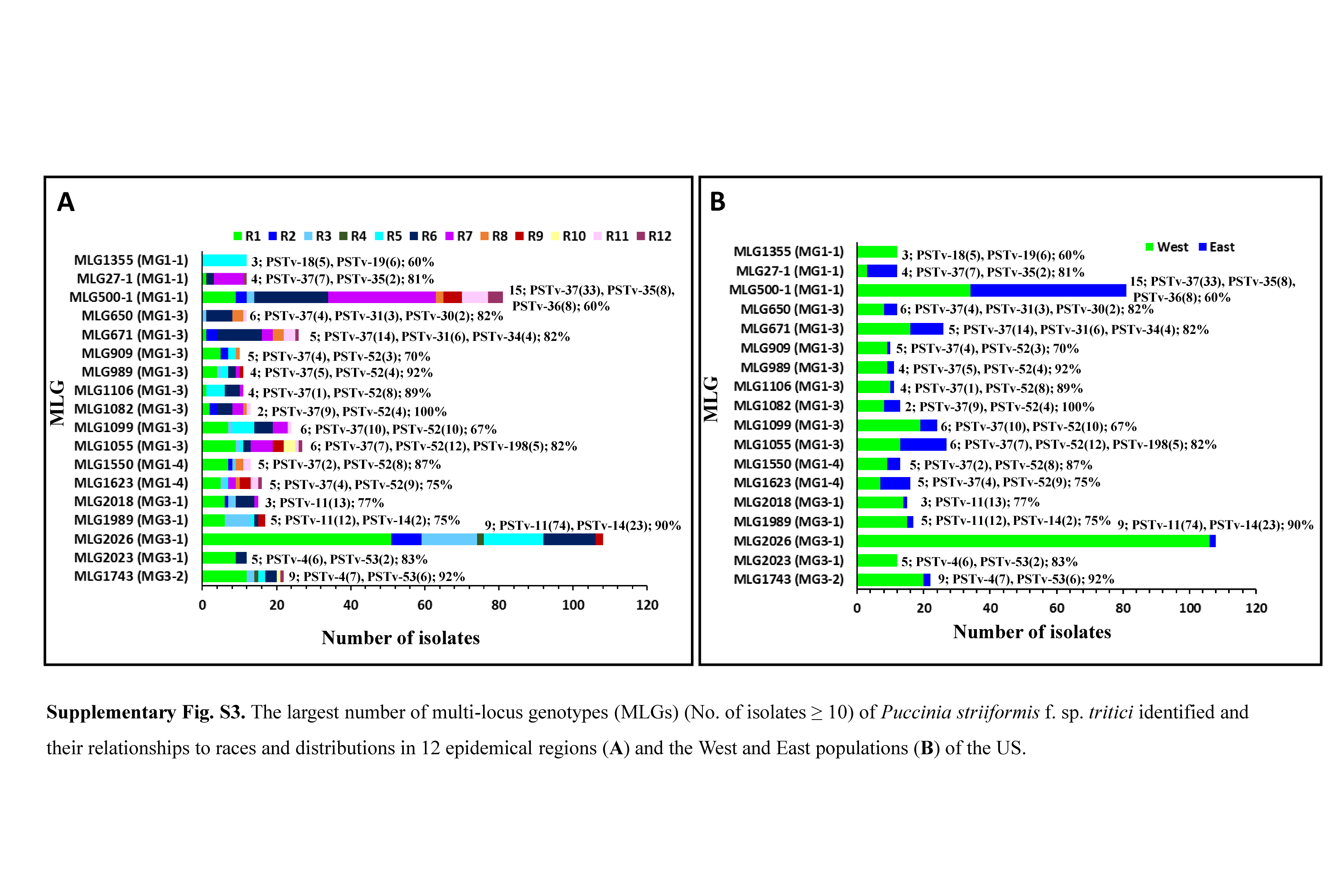

Supplement: Supplementary Figure 3 — The multilocus genotypes (MLGs) with 10 or more isolates of Puccinia striiformis f. sp. tritici and their relationships to races and distributions in 12 epidemical regions (A) and the West and East populations (B) of the United States. [file Image_3.TIF]

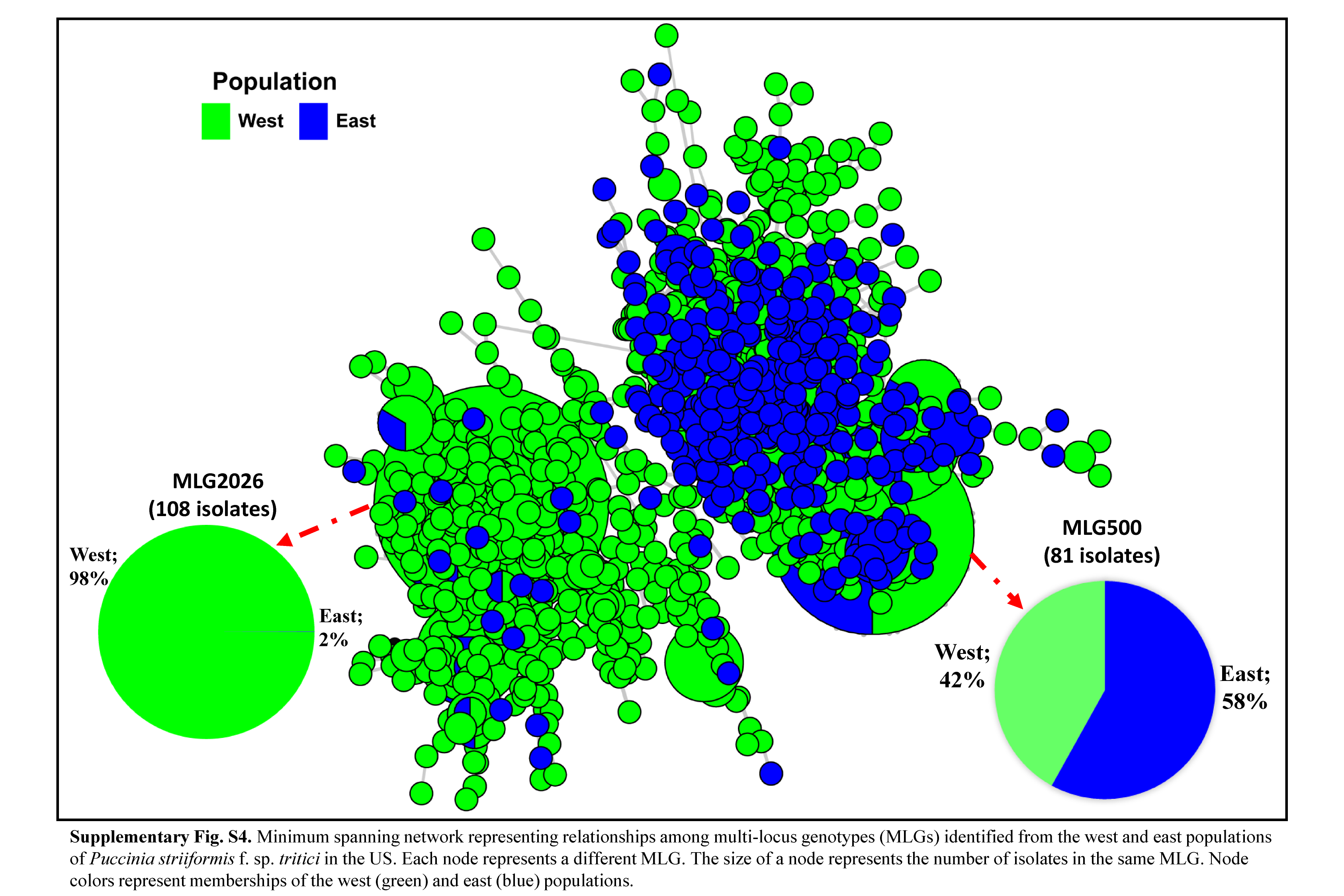

Supplement: Supplementary Figure 4 — Minimum spanning network representing relationships among multilocus genotypes (MLGs) identified from the west and east populations of Puccinia striiformis f. sp. tritici in the United States. Each node represents a different MLG. The size of a node represents the number of isolates in the same MLG. Node colors represent memberships of the west (green) and east (blue) populations. [file Image_4.TIF]

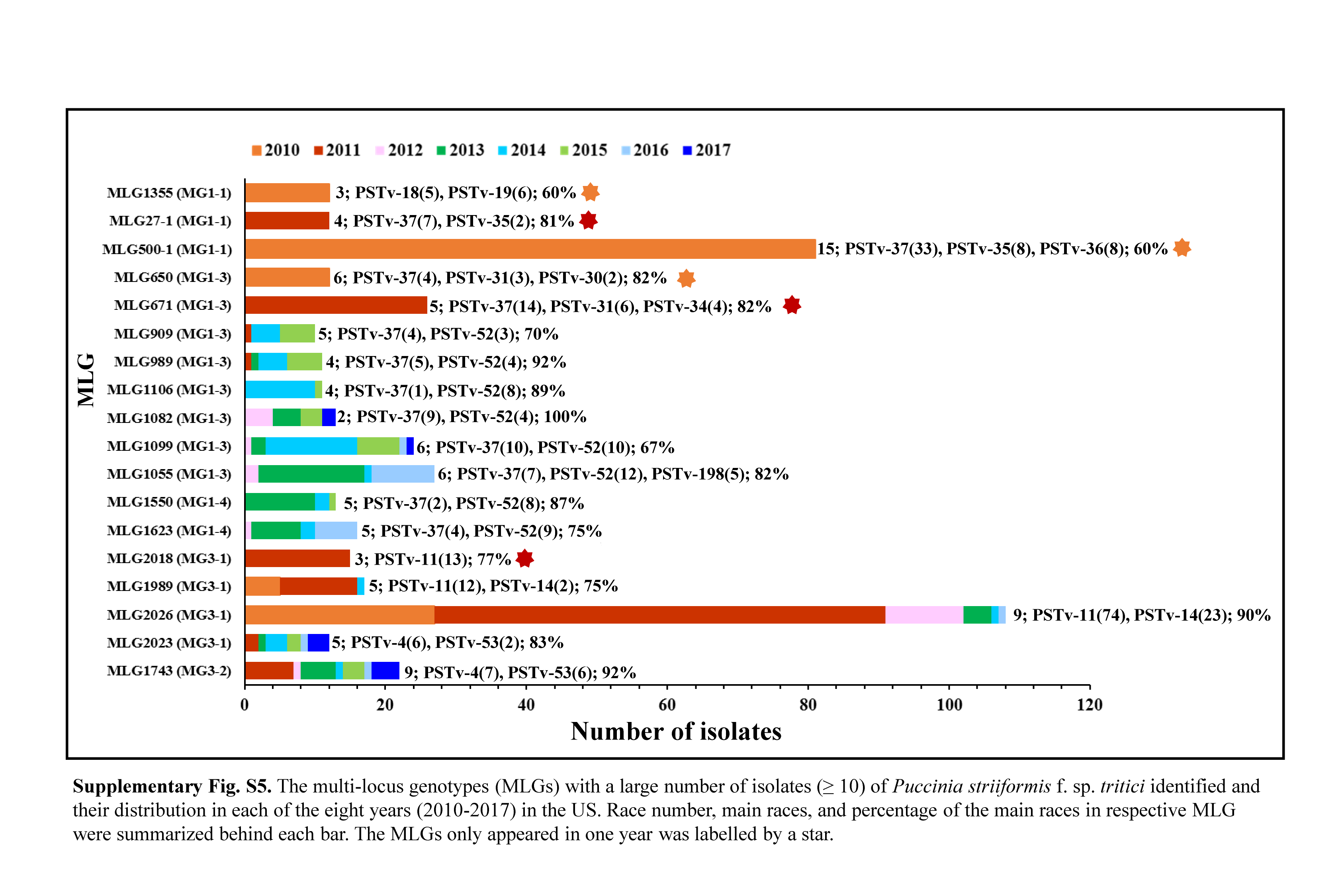

Supplement: Supplementary Figure 5 — Multilocus genotypes (MLGs) with a large number of isolates (≥10) of Puccinia striiformis f. sp. tritici and their distribution in each of the 8 years (2010–2017) in the United States. Race number, main races, and percentages of main races in respective MLG are behind each bar. The MLGs that appeared in only 1 year was labeled by a star. [file Image_5.TIF]

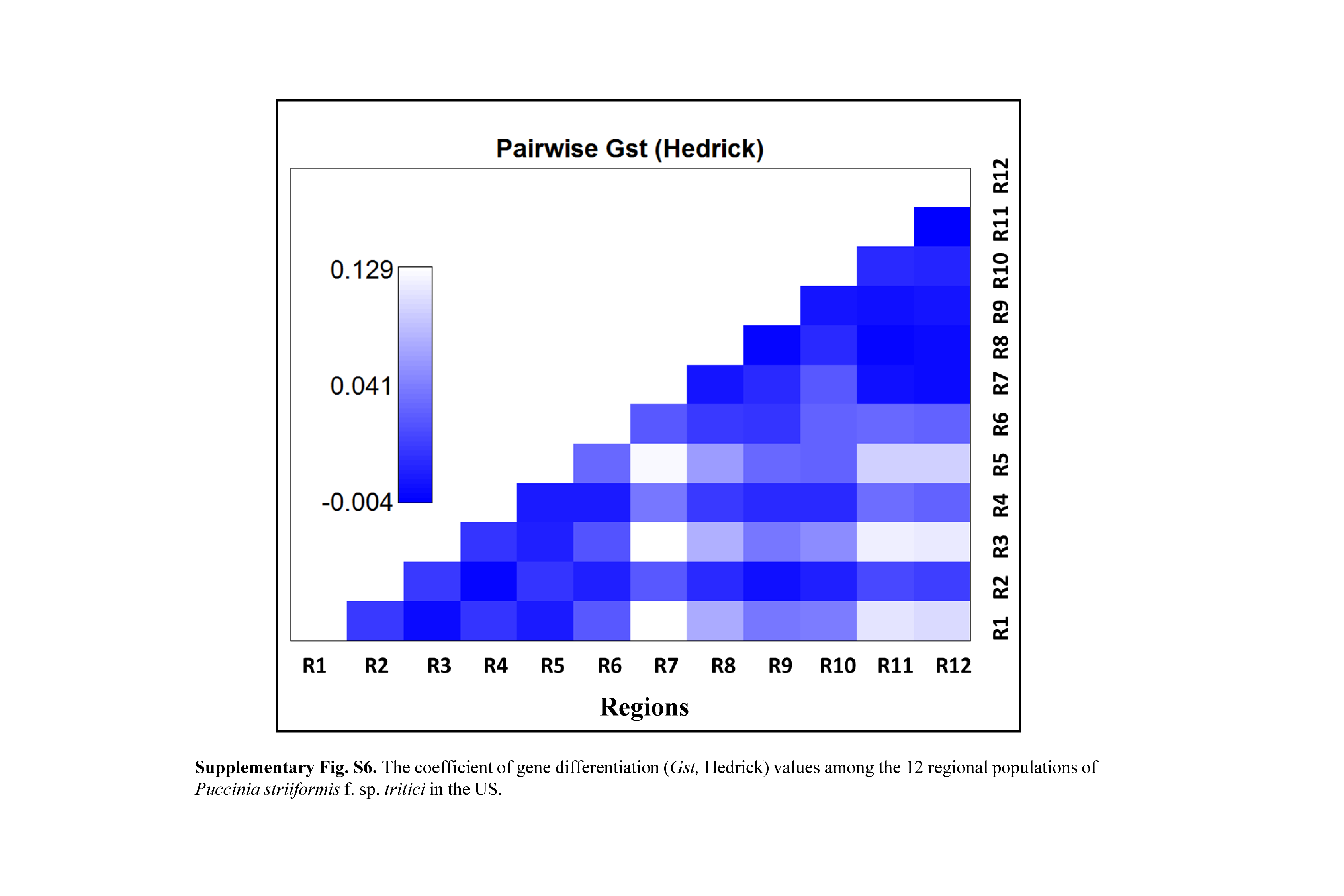

Supplement: Supplementary Figure 6 — Values of gene differentiation (Gst, Hedrick) among the 12 regional populations of Puccinia striiformis f. sp. tritici in the United States. [file Image_6.TIF]

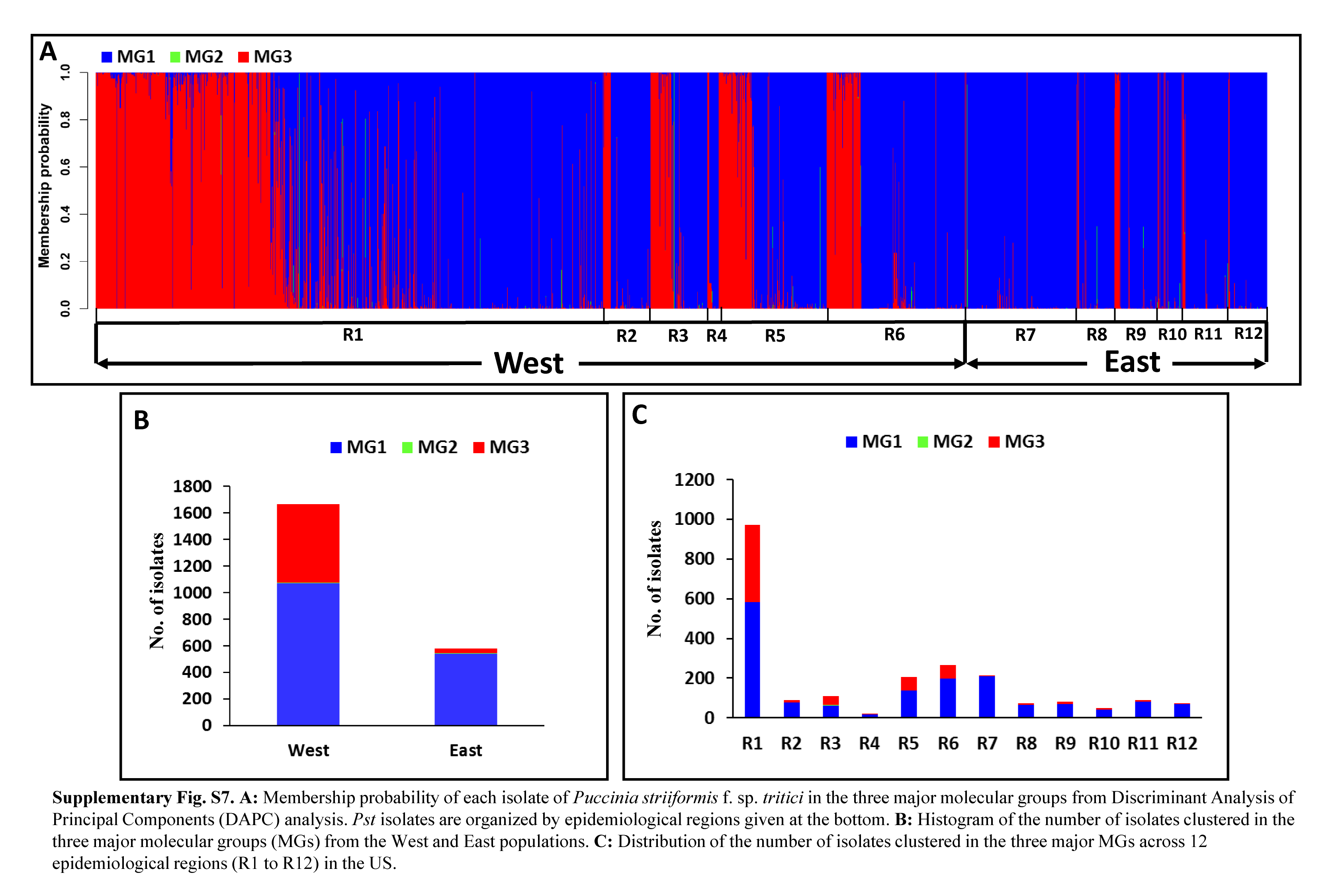

Supplement: Supplementary Figure 7 — (A) Membership probability of each isolate of Puccinia striiformis f. sp. tritici (Pst) in the three major molecular groups from discriminant analysis of principal components (DAPC) analysis. Pst isolates are organized by epidemiological regions given at the bottom. (B) Histogram of the numbers of isolates clustered in the three major molecular groups (MGs) from the West and East populations. (C) Distribution of the numbers of isolates clustered in the three major MGs across 12 epidemiological regions (R1–R12) in the United States. [file Image_7.TIF]

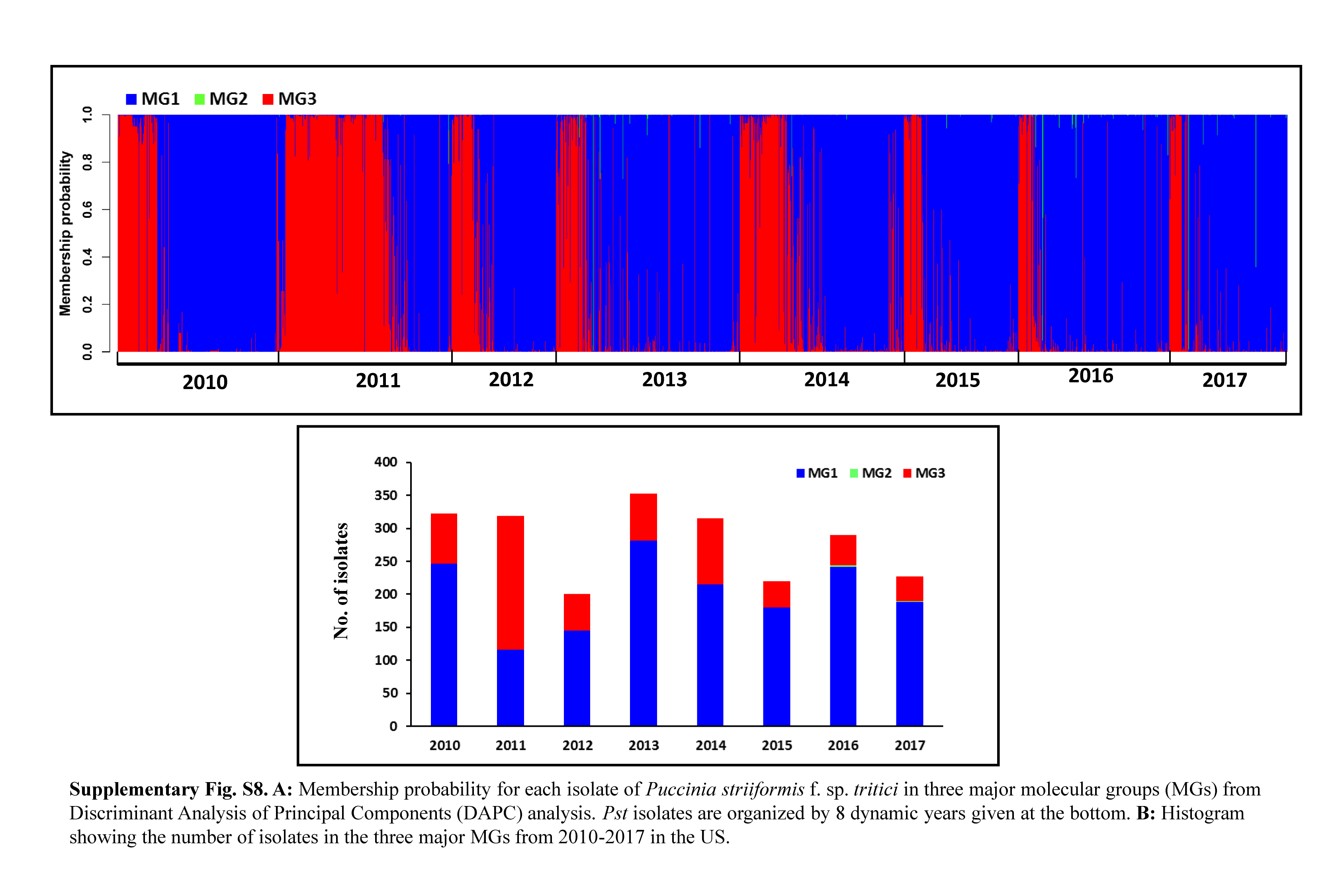

Supplement: Supplementary Figure 8 — (A) Membership probability for each isolate of Puccinia striiformis f. sp. tritici (Pst) in three major molecular groups (MGs) from discriminant analysis of principal components (DAPC) analysis. Pst isolates are organized by 8 years given at the bottom. (B) Histogram showing the numbers of isolates in the three major MGs from 2010–2017 in the United States. [file Image_8.TIF]

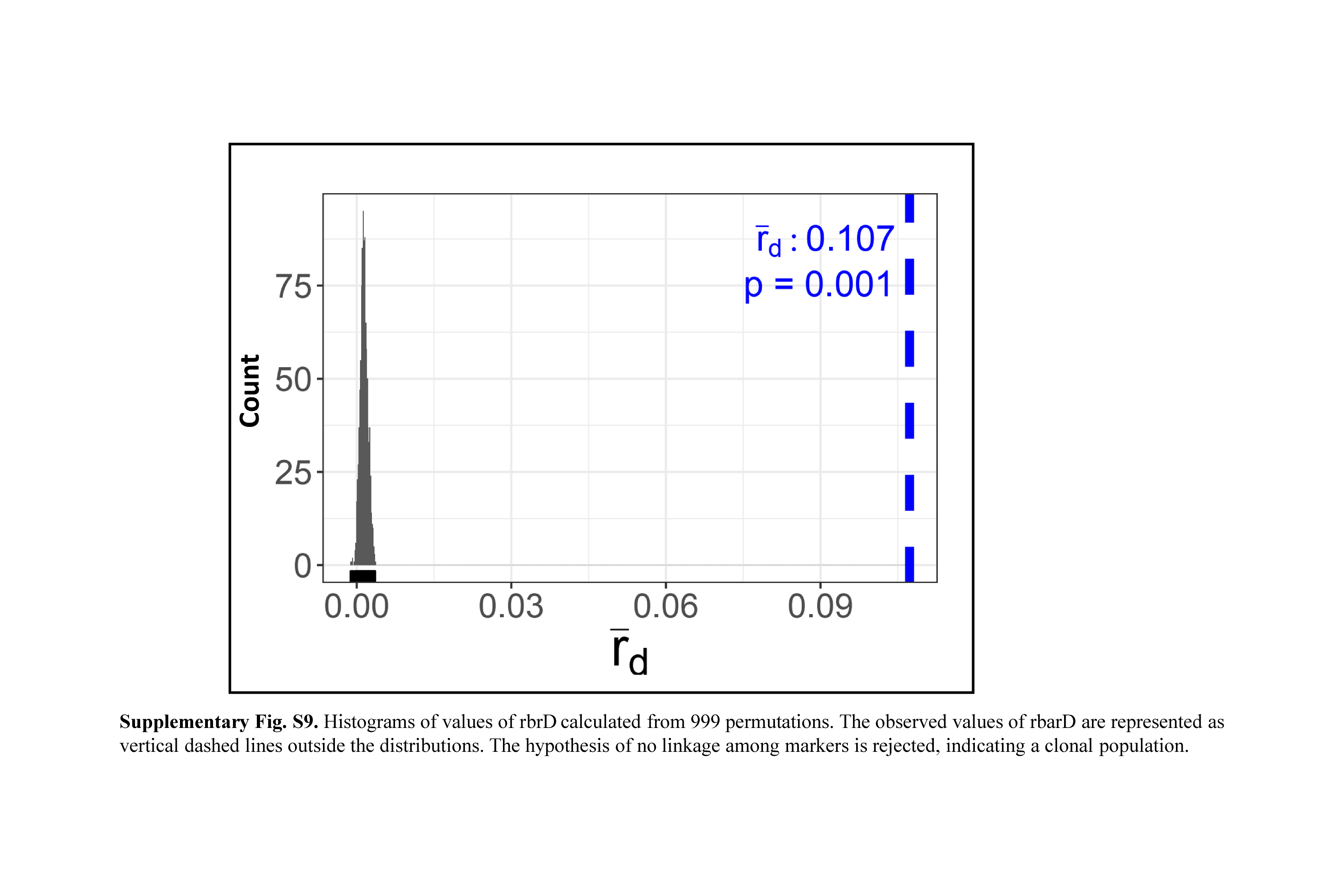

Supplement: Supplementary Figure 9 — Histograms of values of rbrD calculated from 999 permutations. The observed values of rbarD are represented as vertical dashed lines outside the distributions. The hypothesis of no linkage among markers is rejected, indicating a clonal population. [file Image_9.TIF]
